# Trend of malaria parasites infection in Ethiopia along an international border: a Bayesian spatio-temporal study

**DOI:** 10.1186/s40249-025-01320-w

**Published:** 2025-07-11

**Authors:** Changkuoth Jock Chol, Denekew Bitew Belay, Haile Mekonnen Fenta, Ding-Geng Chen

**Affiliations:** 1 Department of Statistics, College of Science, Bahir Dar University, Bahir Dar, Ethiopia; 2 Department of Statistics, College of Natural and Computational Science, Gambella University, Gambella, Ethiopia; 3 Department of Statistics, University of Pretoria, Pretoria, South Africa; 4 Center for Environmental and Respiratory Health Research, Population Health, University of Oulu, Oulu, Finland; 5 Biocenter Oulu, University of Oulu, Oulu, Finland; 6 College of Health Solutions, Arizona State University, Arizona, USA

**Keywords:** Malaria, Bayesian, Integrated nested Laplace approximation, Parasites, Ethiopia, International border

## Abstract

**Background:**

Malaria is a major worldwide health concern that impacts many individuals worldwide. *P. falciparum* is Africa’s main malaria cause. However, *P. vivax* share a large number in Ethiopia than any other countries in Africa, followed by the closest countries. This research aims to examine the spatiotemporal trends in the risk of malaria caused by *P. falciparum* and *P. vivax* in Ethiopia and other countries that share borders between 2011 and 2020.

**Methods:**

This study was carried-out in seven East African countries in 115 administration level 1 (region) settings. We used secondary data on two *plasmodium* parasites, *P. falciparum*, and *P. vivax*, between 2011 and 2020 from the Malaria Atlas Project. This study used a Bayesian setup with an integrated nested Laplace approximation to adopt spatiotemporal models.

**Results:**

We analyzed *P. falciparum* and *P. vivax* malaria incidence data from 2011 to 2020 in 115 regions. Between 2011 and 2020, all of South Sudan's areas, Ethiopia's Gambella region, and Kenya’s Homa Bay, Siaya, Busia, Kakamega, and Vihita regions were at a higher risk of contracting *P. falciparum* malaria than their neighbors in seven East African nations. However, the Southern Nations, nationalities, and people, as well as the Oromia, Harari, Afar, and Amhara areas in Ethiopia, and the Blue Nile in Sudan, are the regions with a higher risk of *P. vivax* malaria than their bordering regions. For both *P. falciparum* and *P. vivax*, the spatially coordinated main effect and the unstructured spatial effect show minimal fluctuation across and within 115 regions during the study period. Through a random walk across 115 regions, the time-structured effect of *P. falciparum* malaria risk shows linear increases, whereas the temporally structured effect of *P. vivax* shows increases from 2011 to 2014 and decreases from 2017 to 2020.

**Conclusions:**

The global malaria control and eradication effort should concentrate particularly on the South Sudan and Ethiopia regions to provide more intervention control to lower the risk of malaria incidence in East African countries, as both countries have high levels of *P. falciparum* and *P. vivax*, respectively.

**Graphical Abstract:**

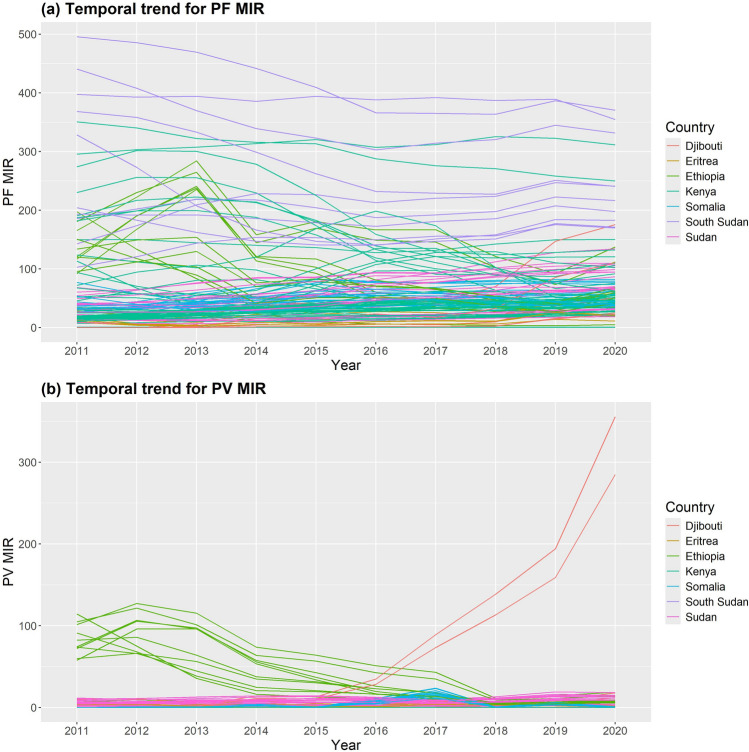

**Supplementary Information:**

The online version contains supplementary material available at 10.1186/s40249-025-01320-w.

## Background

Malaria is a serious global health issue that affects a large number of people globally. Malaria claimed the lives of approximately 627,000 individuals in 2020, with an estimated 241 million cases occurring primarily in global [1]. Infection with single-celled parasites from the *Plasmodium* genus causes malaria. Human sickness is known to be caused by five species of *Plasmodium*: *P. falciparum, P. vivax, P. ovale, P. malariae,* and P. *knowlesi. P. falciparum* and *P. vivax* cause the vast majority of malaria cases around the world. *P. vivax* is the most prevalent malaria parasite, causing severe, even fatal infections and contributing considerably to global morbidity and mortality, while *P. falciparum* is the deadliest.

Different evolutionary lineages, or clades, comprise the *Plasmodium* species that infect humans; these lineages independently produced human parasites that recently shared common ancestors with other nonhuman primate species [2, 3]. Unsurprisingly, at practically every stage of their life cycles, the five parasites that cause malaria in humans exhibit biological variations [4, 5]. The ecological settings and geographic ranges of the parasite species that can infect humans differ and some regions may have a dominating species or all of them [2, 6, 7].

*P. falciparum* is the main cause of malaria infection in Sub-Saharan Africa (SSA) countries, whereas Ethiopia has a higher number of *P. vivax* than any SSA countries, followed by nearest East African countries [8, 9]. Even though the human *P. vivax* parasite is thought to have originated in Africa [10], its presence on the continent has been unevenly distributed, and its clinical effects are regarded as being minimal outside of Eastern Africa [11]. South Sudan, the island of Madagascar, and the Horn of Africa (Ethiopia, Djibouti, Eritrea, and Somalia) appear to be the only countries where *P. vivax* is endemic and consistently causes serious clinical illness. However, reports from numerous other African nations attest to the parasite’s widespread distribution.

Given that this species is believed to require the Duffy receptor to invade reticulocytes and cause disease, the higher prevalence of Duffy-positive individuals in these countries (and its widespread absence in the rest of the continent) is likely linked to such disparate distribution of clinical disease [12]. Nonetheless, over the last decade, there has been an increase in the number of *P. vivax*-related infections and illnesses in Duffy-negative people from diverse West African countries [13, 14]. This supports the species'widespread presence in other malaria-endemic areas of Africa and raises the possibility that *P. vivax* has evolved to infect reticulocytes in different ways and cause disease [15]. Even though this condition is uncommon, it may make it more difficult to meet the continent's current malaria elimination targets [14]. The parasite populations of East Africa are widely recognized to form a genetic cluster when compared to those of other regions of Africa. East African parasite groups'close closeness to one another typically facilitates their transmission relationship and strong genetic ancestry [16–18].

The two primary methods for managing mosquito populations that transmit malaria are indoor residual spraying (IRS) and insecticide-treated mosquitoes (ITNs). The use of IRS has decreased after 2000, whereas the use of ITN has grown rapidly. In SSA, ITNs serve as the cornerstone of efforts to control malaria [1]. The proportion of pregnant women and children under five who slept under an ITN rose from 3 to 49% between 2000 and 2020. Between 2000 and 2020, 43% of the overall at-risk population slept under an ITN, up from 2% in 2000. In SSA, the overall utilization of ITNs has somewhat decreased since 2017. IRS is the process of applying insecticides to a home’s interior walls and ceilings in order to eradicate mosquitoes that come into contact with them. The IRS-protected population fell from 5.8% in 2010 to 2.6% in 2020 on a global scale. During the same time period, Africa's protected population fell from 11.2% to 5.3%. Those covered by IRS worldwide decreased from 161 million in 2010 to 127 million in 2015 and then to 87 million in 2020 [1]. Between 2010 and 2020, manufacturers sold 3.1 billion RDTs worldwide, with SSA accounting for almost 81% of sales. 2.2 billion RDTs were provided during that time by national malaria programs, with 88% of those distributions occurring in sub-Saharan Africa [1].

The World Health Organization's (WHO) Global Malaria Programme (GMP) High Burden High Impact (HBHI) strategy urges nations to employ a variety of data sources to provide them with a better understanding of the sub-national malaria risk. To optimize the impact of malaria control and make the most of limited resources, these national epidemiological frameworks ought to be employed [1, 19]. To promote health equity, data should ideally be stacked to give meaningful stratification based on the epidemiology of malaria risk and burden, vulnerability, marginalization, and locations with inadequate intervention coverage. To direct sub-national stratification, policies, and resource allocation, National Malaria Control Programmed (NMCPs) require that information be resolved in administrative areas [20, 21]. Accurate diagnosis of every *P.* species is necessary for elimination and eradication efforts. *P. malariae*, *P. ovale* (*P. ovale curtisi* and *P. ovale wallikeri*), and *P. vivax* have been documented. Nevertheless, *P. falciparum* is the most common species in SSA [22].

Using highly specific technologies such as Next Generation Sequencing (NGS) could exceed the detection limit of current malaria diagnosis tools, which are prone to missing low-density *parasitemia*, which is common in silent malaria infection at the community level. Sequencing enables the detection of both *P. falciparum* and *non-falciparum* species at all *parasitemia* levels, as well as the capture of new cases undetected by the current surveillance system. More precise statistics could disclose the true malaria burden and inform measures for malaria control, elimination, and, eventually, eradication. This study proposes a Bayesian spatiotemporal analysis of malaria parasite infection in Ethiopia and other international border nations between 2011 and 2020.

## Methods

### Settings

**Fig. 1:**
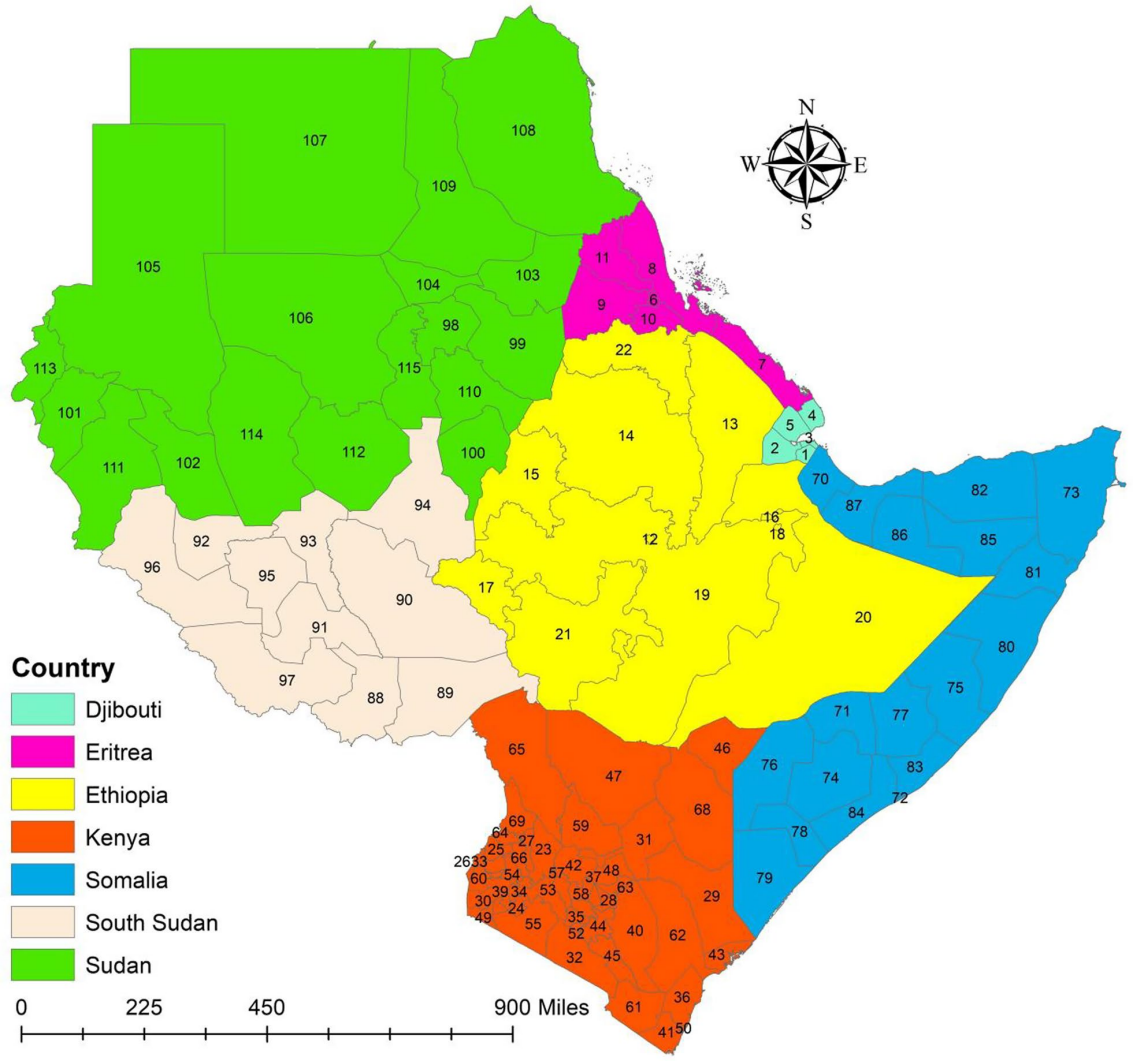
Map of the study area. Source of shapefile: Database of Global Administrative Areas v.4.1 (www.gadm.org), own map output from ArcGIS (v.10.8)

This study was carried out in Ethiopia and the nations that share an international border with it. Ethiopia is in the Horn of Africa region and borders six countries: Somalia, Sudan, South Sudan, Eritrea, Kenya, and Djibouti (Fig. 1). We conducted this study in 115 administration level 1 (sub-national) settings across seven East African countries. For the purposes of this study, the seven nations are all referred to as regions, even if they have different names for administration level 1. The name of the regions and data of the study area were provided in the supplemental file (Table S2 and Table S3).

### Data sources

The data used in this study are secondary data sources gathered from the Malaria Atlas Project (MAP) website (https://malariaatlas.org) [23]. Between 2011 and 2020, we used data on malaria cases caused by two *plasmodium* parasites, *P. falciparum* and *P. vivax*, in 115 regions across seven countries in SSA. The MAP is a collaborative research and advocacy platform that provides complete and current information on malaria distribution, burden, and control. For more than ten years, the MAP has worked to create and maintain a global open-access database of spatial *malariometric* data. At the global, regional, national, subnational (in our case, region), and 5-square-kilometer levels, MAP examines the risk and burden of two *Plasmodium* parasites. We downloaded *P*. *falciparium* and *P. vivax* data at subnational levels from MAP in 115 regions across 7 East African nations in excel file format. This data comes in a variety of formats and themes, including geo-located surveys of malaria parasite rates, global administrative boundary shapefiles, and global and regional raster depicting the distribution of malaria and related illnesses, blood disorders, and intervention coverage [24]. We used shape files from the Database of Global Administrative Areas dataset version 4.1 (www.gadm.org) to aggregate data by averaging at the first administrative level within 115 regions in the seven East African countries between 2011 and 2020. We used UN population estimates [25] to calculate the number of people at risk of malaria. The number of malaria cases in the population at risk each year is known as the malaria case count. People who reside in malaria-infested areas are considered part of the at-risk population.

### Statistical analytic models

#### Testing for spatial autocorrelation and detecting malaria cluster

It is crucial to implement or even create techniques that can accurately and consistently identify malaria clusters in both geography and time in order to ascertain the spatial pattern of malaria clustering. Numerous methods, including Anselin Local Moran's *I* statistic (local indicators of spatial association [LISA]) [26], Getis-Ord Gi* statistics [27], SaTScan [28], and Flex Scan [29], have been used to identify spatial and space–time clusters.

Spatial scan statistics, particularly SaTScan version 10.0, CDC Foundation, New York City, USA and Flex Scan 600 PPM, nextScan, 719 N. Principle Place, Suite 130|Meridian, ID 83642, have been employed in a wide range of epidemiological investigations and disease surveillance. However, it does not appear to be well known that these spatial scan statistics, particularly SaTScan, tend to find the most likely cluster, which is considerably larger than the true cluster, by absorbing nearby regions with a low likelihood of disease occurrence. As a result, if researchers revealed the observed most likely cluster as is, they may face criticism because it includes some places with no higher risk [30].

In this study, we proposed LISA and the Getis-Ord Gi* statistics to prevent finding such undesired and misleading clusters that may generate public worry. The Moran's *I* statistic was used to investigate spatial autocorrelation on a global scale, while the LISA and the Getis-Ord Gi* statistics were used to quantify it on a local scale. The global Moran's *I* statistic was used to analyze the presence and intensity of spatial autocorrelation over the entire study area, as well as to test the spatial independence assumption utilized in the spatial pattern analysis. The LISA and Getis-Ord Gi* statistics were used to detect malaria clustering and locate hotspots. These investigations were carried out using capabilities available in ArcMap software version 10.8, developed by Environmental Systems Research Institute, Inc. For further information on spatial autocorrelation statistics, refer to the supplemental file (Text S1).

#### Spatiotemporal models

Spatiotemporal disease mapping models are widely used in disease surveillance studies where the interest is to identify the spatial and temporal pattern of a disease [31, 32]. In this study, we used counts of *P. falciparum* and *P. vivax* malaria incidence for 115 regions in the 7 East African countries of SSA during 2011 − 2020 to build a space–time disease mapping. We are interested in investigating the *P. falciparum* and *P. vivax* malaria risk in 115 regions from 2011 to 2020.

Let ${y}_{it}$ be the *P. falciparum* or *P. vivax* malaria cases in the ${i}^{th}$ region at time$t$, and ${n}_{it}$ be the size of the corresponding population at risk, for ${\text{i}}\left( {{\text{i}} = 1,2, \ldots ,45} \right)$ and ${\text{t}}\left( {{\text{t}} = 2011, \ldots ,2020} \right)$. For the ${i}^{th}$ area and time$t$, the number of *P. falciparum* or *P. vivax* malaria incidence ${y}_{it}$ is modeled as

$${y}_{it}\sim Po({\lambda }_{it})$$

where the mean ${\lambda }_{it}$ is defined in terms of a rate ${\rho }_{it}$ and the expected number of *P. falciparum* or *P. vivax* malaria incidence ${E}_{it}$. In this case, the linear predictor is defined on the logarithmic scale

1 $${\eta }_{it}=log({\rho }_{it})=\alpha +{u}_{i}+{v}_{i}+{Temporal}_{t}$$

with$t=2011, ..., 2020$. On ${Temporal}_{t}$ a parametric or non-parametric structure can be specified. Here, α represents the overall risk in the East African countries, $\mu_{i}$ is a random effect specific to area $i$ to model spatial dependence between risks, and ${v}_{i}$ is an unstructured exchangeable component that models uncorrelated noise, $v_{i} \sim N\left( {0, \sigma_{v}^{2} } \right)$.

##### Parametric trend

The classical parametric formulation was introduced by Bernardinelli et al. (1995) [33], and assume that the linear predictor can be written as

2 $$\eta_{it} = \alpha + u_{i} + v_{i} + \left( {\beta + \delta_{i} } \right) \times t$$

Here $\alpha$ is the intercept quantifying the average *P. falciparum* or *P. vivax* malaria incidence rate in all 115 regions, $\xi_{i} = u_{i} + v_{i}$ is the spatial and random effect of the areas; a main linear trend $\upbeta$, which represents the global time effect; and a differential trend ${\delta }_{i}$, which identifies the interaction between time and space.

We assume a Besag-York-Mollie (BYM) specification [34], so ${u}_{i}$ is the spatially structured residual, modeled using an intrinsic conditional autoregressive structure (iCAR)

$${u}_{i}|{u}_{j\ne i}\sim N\left(\frac{\sum_{j\epsilon n(i)}{u}_{i}}{N(i)}, {s}_{i}^{2}=\frac{{\sigma }^{2}}{N(i)}\right),$$

where $\text{N}(\text{i})$ is the number of regions that share boundaries with the ${i}^{th}$ region (i.e. its neighbour), as presented in Banerjee et al. (2017) [35]. The parameter ${v}_{i}$ represents the unstructured residual, modeled using an exchangeable prior: $v_{i} \sim N\left( {0, \sigma_{v}^{2} } \right)$. Since, for identifiability purposes, a sum-to-zero constraint is imposed on $\updelta$ and$\text{v}$, the terms ${\delta }_{i}$ represent the difference between the global trend $\upbeta$ and the area-specific trend. If ${\delta }_{i}<0$ then the area-specific trend is less steep than the mean trend, whilst ${\delta }_{i}>0$ implies that the area-specific trend is steeper than the mean trend. We assume $\delta_{i} \sim N\left( {0, 1/\tau_{\delta } } \right)$, but other specifications can be used, e.g. a conditional autoregressive structure [33].

##### Non-parametric dynamic trend

Every component in the model put forward by Bernardinelli et al. (1995), is assumed to have a linear temporal trend [33]. Alternative models that do not require linearity and assume a non-parametric model for the time trend have also been proposed [36]. The assumption of linearity in the ${\delta }_{i}$ can be released [37], using a dynamic non-parametric formulation for the linear predictor

3 $${\eta }_{it}=\alpha +{u}_{i}+{v}_{i}+{\gamma }_{t}+{\phi }_{t}$$

Here $\alpha \,u_{i}$ and $v_{i}$ have the same parametrization as in (2); however, the term ${\gamma }_{t}$ represents the temporal structured effect, modeled dynamically (in our case, using a random walk-in time of second order) through a neighboring structure:

$${\upgamma }_{{\text{t}}} |{\upgamma }_{{{\text{t}} - 1}} ,{\upgamma }_{{{\text{t}} - 2}} \sim {\text{N}}\left( {2{\upgamma }_{{{\text{t}} - 1}} - {\upgamma }_{{{\text{t}} - 2}} ,{\upsigma }_{{\upgamma }}^{2} } \right)$$

Finally, ${\phi }_{t}$ is specified using a Gaussian exchangeable prior:$\phi_{t} \sim N\left( {0, 1/\tau_{\phi } } \right)$

##### Space–time interaction

It is easy to expand the non-parametric dynamic trend model to allow for an interaction between space and time, which would explain differences in the time trend of *P. falciparum* or *P. vivax* malaria incidence to different areas, e.g. using the following specification:

4 $${\eta }_{it}=\alpha +{u}_{i}+{v}_{i}+{\gamma }_{t}+{\phi }_{t}+{\delta }_{it}$$

Here$\alpha ,\mu_{i} ,\,and\,\,v_{i}$ have the same parametrization as in (2), whereas ${\gamma }_{t}$ and ${\phi }_{t}$ have the same parametrizations as in (3). The parameter vector $\delta$ follows a Gaussian distribution with a precision matrix given by ${\tau }_{\delta }{\rm H}_{\delta }$, where ${\tau }_{\delta }$ is the unknown scalar, and ${\rm H}_{\delta }$ is the structure matrix, identifying the types of temporal and spatial dependence between the element of $\delta$. ${\rm H}_{\delta }$ may be factorized as the Kronecker product of the structural matrix of the respective primary effects that interact, by Clayton (1996) [38]. There are four ways to define the structure matrix, as presented in Knorr-Held (2000) [37].

The type I interaction assumes that the two unstructured effects $${v}_{i} and {\phi }_{t}$$ interact. We write the structure matrix as $${\rm H}_{\delta }={\rm H}_{v}\otimes {\rm H}_{\phi }=I\otimes I=I$$, because both $$v$$ and $$\phi$$ do not have a spatial and temporal structure. Consequently, we assume no spatial and/or temporal structure on the interaction either and therefore, $$\delta_{it} \sim Normal(0,\sigma_{\delta }^{2} = {\raise0.7ex\hbox{$1$} \!\mathord{\left/ {\vphantom {1 {\tau_{\delta } }}}\right.\kern-0pt} \!\lower0.7ex\hbox{${\tau_{\delta } }$}}$$. The type II interaction combines the structured temporal main effect $${\gamma }_{t}$$ and unstructured spatial effect$${v}_{i}$$. We write the structure matrix as$${\rm H}_{\delta }={\rm H}_{v}\otimes {\rm H}_{\gamma }$$, where $${\rm H}_{v}=I$$ and $${\rm H}_{\gamma }$$ is the neighborhood structure specified for instance through a first-or-second random walk (RW). This leads to the assumption that for the $${i}^{th}$$ area, the parameter vector $$\{{\delta }_{i1},\dots ,{\delta }_{it}\}$$ has an autoregressive structure on the time component, which is independent of the ones of the other areas. The matrix $${\boldsymbol{\rm H}}_{\delta }$$ has a rank of $$n(T-1)$$ for a first-order RW and $$n(T-2)$$ for a second-order RW. The type III interaction combines the unstructured temporal effects $${\phi }_{t}$$ and the spatially structured main effect$${u}_{i}$$. We write the structure matrix as$${\boldsymbol{\rm H}}_{\delta }={\boldsymbol{\rm H}}_{\phi }\otimes {\boldsymbol{\rm H}}_{u}$$, where $${\boldsymbol{\rm H}}_{\phi }={\varvec{I}}$$ and $${\boldsymbol{\rm H}}_{u}$$ is the neighboring structure defined through the CAR specification. This leads to the assumption that the parameters of the $${t}^{th}$$ time point $$\{{\delta }_{1},\dots ,{\delta }_{n}\}$$ have a spatial structure independent from the other time points. The matrix $${\boldsymbol{\rm H}}_{\delta }$$ has a rank of$$T(n-1)$$. Type IV interaction is the most complex type of interaction, assuming that the spatially and temporally structured effects $${u}_{i}$$ and $${\gamma }_{t}$$ interact. The structure matrix can be written as the *Kronecker* product of $${\boldsymbol{\rm H}}_{\delta }={\boldsymbol{\rm H}}_{u}\otimes {\boldsymbol{\rm H}}_{\gamma }$$ and has a rank of $$(n-1)(T-1)$$ for a RW of order 1, and of $$(n-1)(T-2)$$ for a RW of order 2.

##### Estimation methods and model comparison

R-INLA in R software (Version 4.4.2) was used in this work for the model parameter and hyper-parameter estimation since it provides Bayesian estimates faster than Markov Chain Monte Carlo Methods (MCMC) [39]. In this study, minimally informative priorities are specified. In the six models (parametric trend, non-parametric dynamic trend, and four space–time interaction), we assume specification of R-INLA for the distribution of the hyper-parameters; therefore, minimally informative priors are specified on the $log$ of the structured effect precision $log{\tau }_{u}\sim logGamma(1, 0.001)$ and the $log$ of the unstructured effect precision $log{\tau }_{v}\sim logGamma(1, 0.001)$. In addition, we specify a $Gamma(1, 0.001)$ prior the precision of the random walk and of the two unstructured and two structured effects. We defined the precision as $\uptau =1/{\upsigma }^{2}$. To compare the models studied, we use the Deviance Information Criterion (DIC) proposed by Spiegehalter et al. (2002) [40]. The DIC is a generalization of the Akaike information criterion (AIC), which was designed to compare Bayesian models. It consists of two parts: measurements of model fit and an assessment of the model's complexity. This is a criterion that aims to achieve a balance between the adequacy of a model and its complexity. It is defined by $DIC=D+2p$ where $D$ is the posterior mean deviance of the model and $p$ is the effective number of parameters. The model with the smallest value of DIC has a better balance between the model’s adjustment and complexity.

## Results

### Descriptive statistics results

**Fig. 2:**
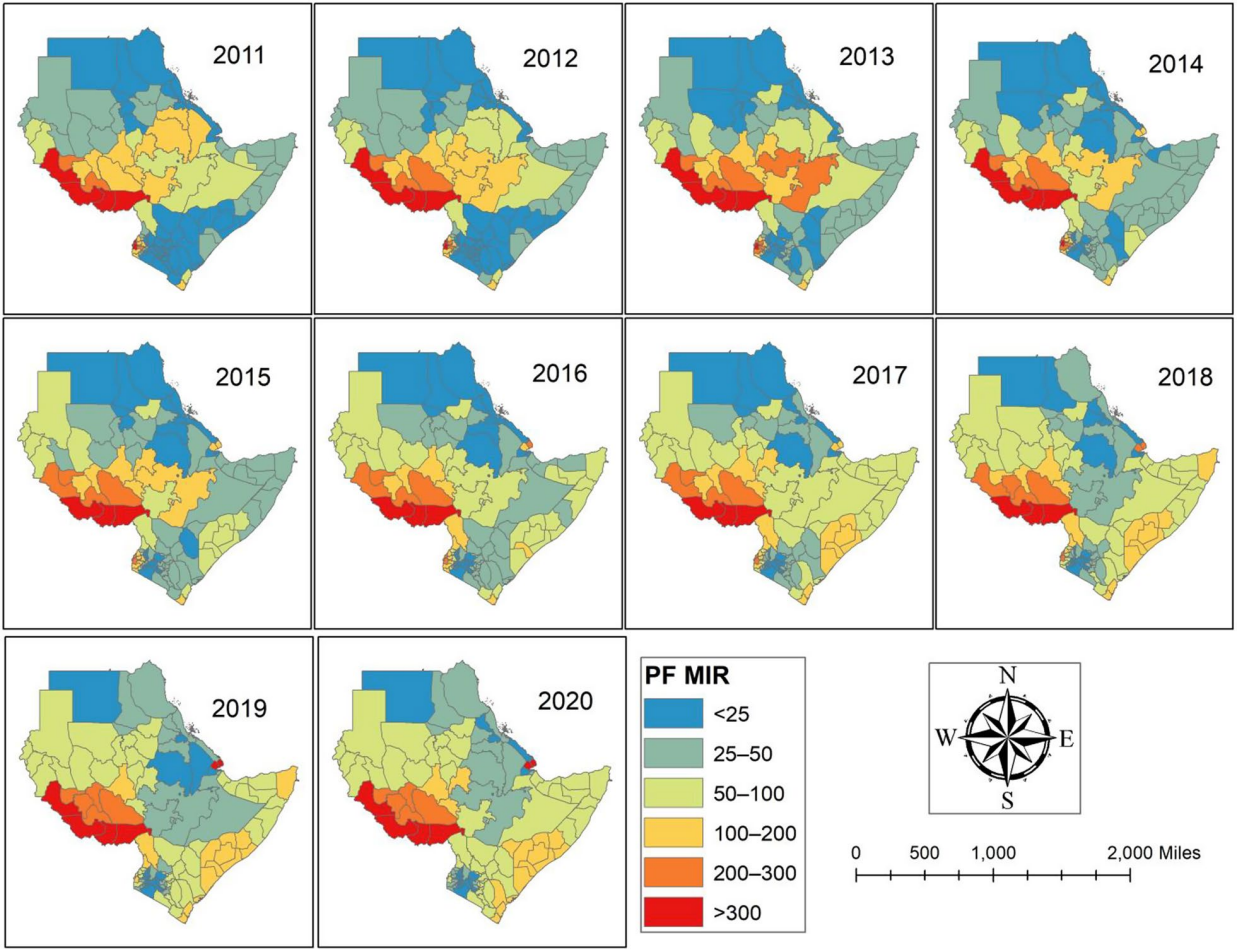
Observed value of the *Plasmodium falciparum* (PF) MIR between 2011 and 2020. Source of shapefile: Database of Global Administrative Areas v.4.1 (www.gadm.org), own map output from ArcGIS (v.10.8). *MIR* Malaria incidence rate

The observed *P. falciparum* malaria incidence rates (MIR) per 1000 people for 115 regions across 7 Eastern African nations between 2011 and 2020 are shown in Fig. 2 and supplemental file (Table S2). The results showed that during the study period, the MIR of *P. falciparum* was highest in the areas around the nation of South Sudan (Table S2). Furthermore, a few areas in Southwest Ethiopia, West Ethiopia, and West Kenya have a high MIR. Additionally, during the study period, the areas around Somalia and Sudan exhibit an increase. However, between 2015 and 2019, the observed value of *P. falciparum* MIR decreased in many Ethiopian spots.

From 2011 to 2020, Fig. 3 and supplementary file (Table S3) showed the estimated value for *P. vivax* MIR per 1000 population, spatially and temporally distributed. The results of the study indicate that there were no cases of *P. vivax* malaria in any of the areas around South Sudan and Kenya during the study period. The areas surrounding Ethiopia have the highest *P. vivax* MIR per 1000 people between 2011 and 2017 (Table S3). Furthermore, compared to other places in the research area, the Obock and Tadjourah regions of Djibouti faced higher MIR caused by *P. vivax* between 2017 and 2020 (Table S3).

**Fig. 3:**
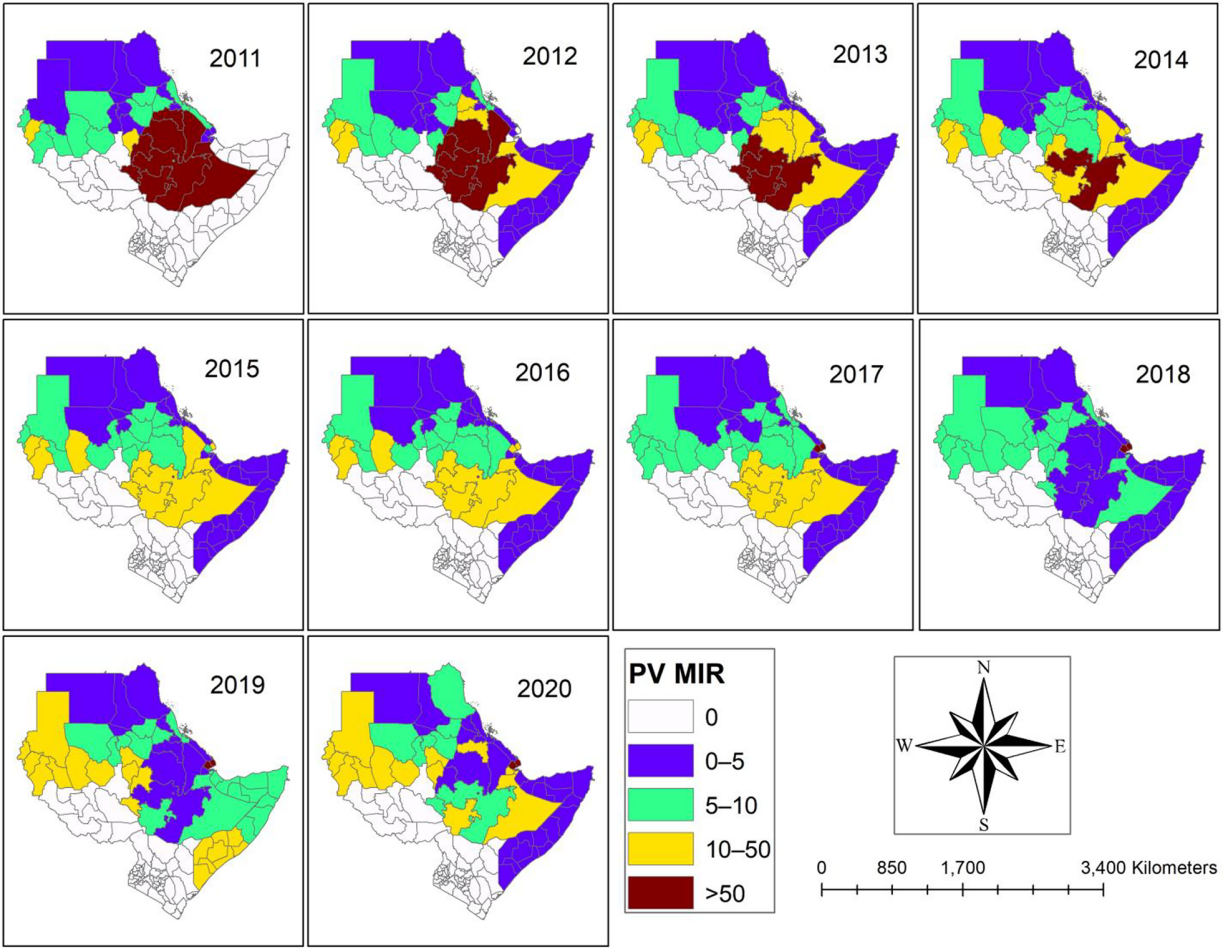
Observed value of the *Plasmodium vivax* (PV) MIR between 2011 and 2020. Source of shapefile: Database of Global Administrative Areas v.4.1 (www.gadm.org), own map output from ArcGIS (v.10.8). *MIR* Malaria incidence rate

**Fig. 4:**
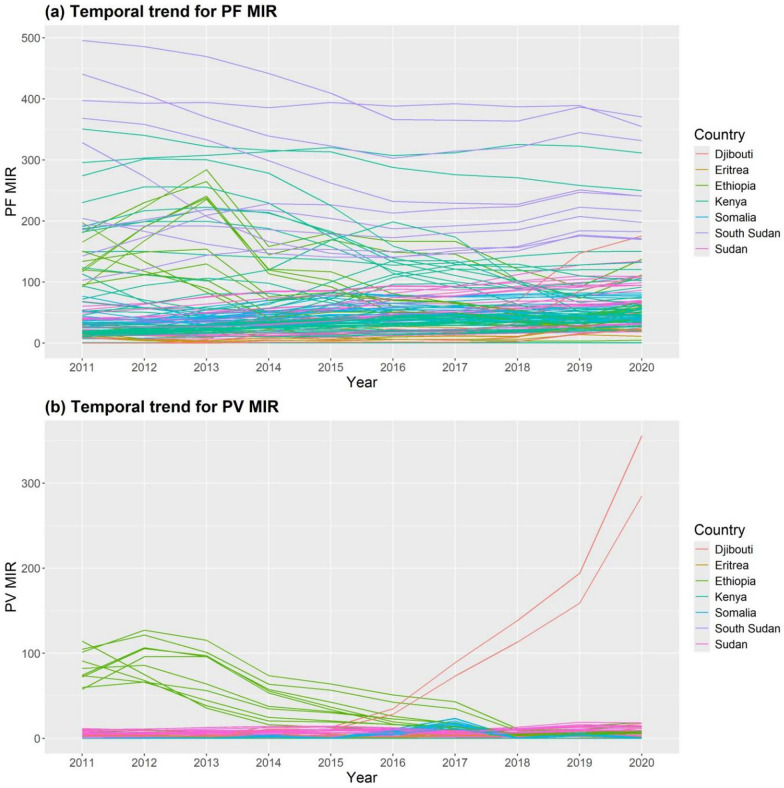
Temporal trend for observed values of yearly MIR in the study area at each region of 115 regions in 7 East African countries of SSA from 2011 to 2020: (**a**) *Plasmodium falciparum* (PF) and (**b**) *Plasmodium vivax* (PV) *MIR* Malaria incidence rate

The results of the temporal trend for the observed values of *P. falciparum* and *P. vivax* MIR per 1000 people between 2011 and 2020 are shown in Fig. 4 (a) and (b), respectively. This research showed that, between 2011 and 2020, the MIR of *P. falciparum* and *P. vivax* varied at random in every region of the study area. Between 2012 and 2020, the recorded levels of *P. vivax* MIR in the areas surrounding Ethiopia decreased. However, the greatest increases in *P. vivax* MIR from 2015 to 2020 are found in Obock and Tadjourah in Djibouti. *Plasmodium vivax* levels were found to have moderately increased in various Somalia regions in 2017. Additionally, during the study period, the *P. vivax* MIR in the areas surrounding Kenya, South Sudan, and Eritrea stays stable.

To determine whether the pattern expressed is clustered, diffused, or random for both *P. falciparum* and *P. vivax* malaria incidence, we estimated the Global Moran's *I*. *P. falciparum* and *P. vivax* Global Moran’s *I* values over the study period were 0.512385 (*z-score* = 12.378825, *P-value* < 0.001) and 0.337284 (*z-score* = 8.428096, *P-value* < 0.001), respectively. These values show a significant spatial clustering of the regions in seven East African countries of the incidence of malaria. The Supplemental file (Table S1) contains the Global Moran's *I* autocorrelation value for the yearly incidence of *P. falciparum* and *P. vivax* malaria cases in East African nations from 2011 to 2020.

**Fig. 5:**
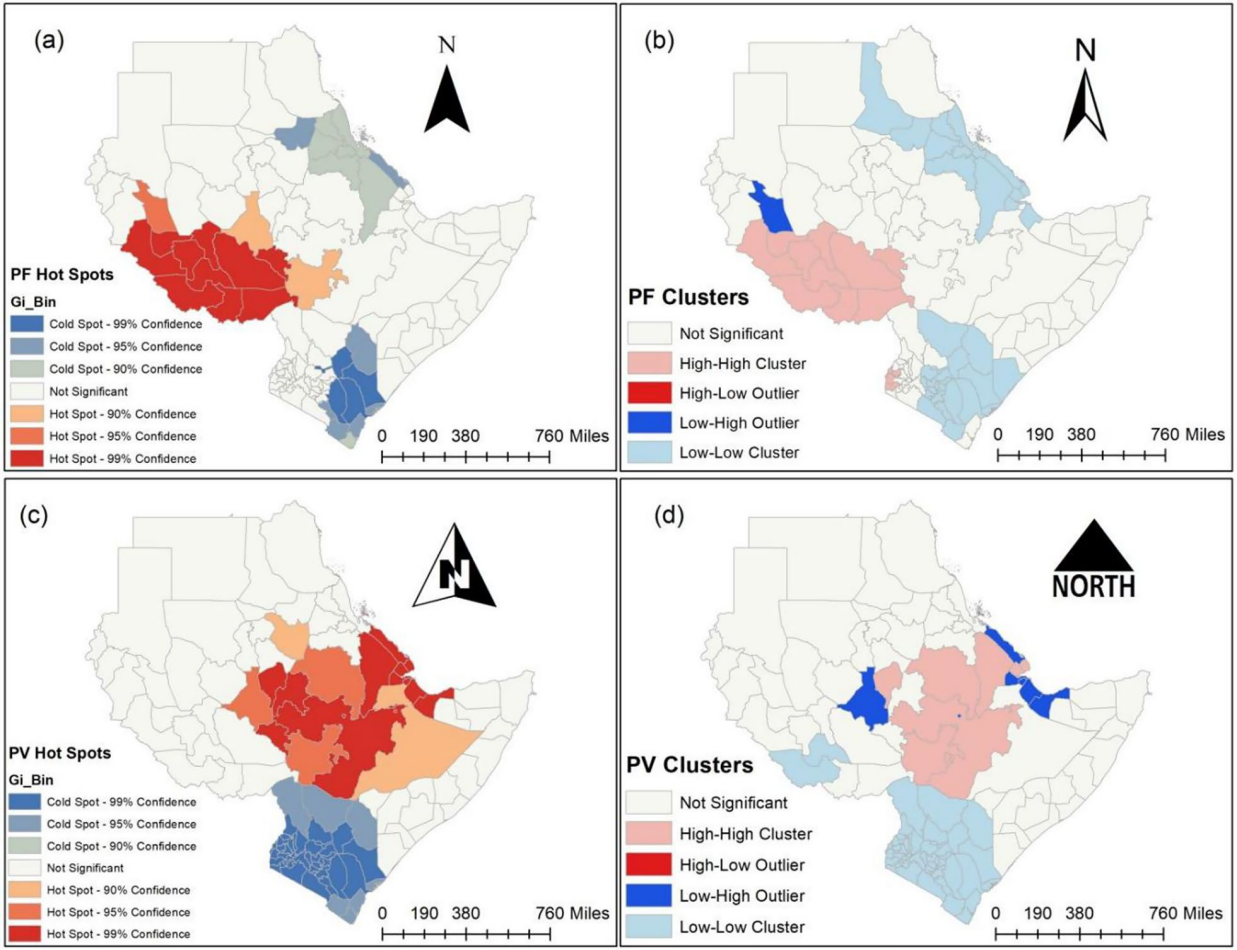
Malaria cluster maps by regions of East Africa, SSA, 2011 − 2020. (**a**) Getis-Ord Gi* statistics and (**b**) Anselin’s Local Moran’s *I* for *Plasmodium falciparum* (PF). **c** Getis-Ord Gi* statistics and (**d**) Anselin’s Local Moran’s *I* for *Plasmodium vivax* (PV). Source of shapefile: Database of Global Administrative Areas v.4.1 (www.gadm.org), own map output from ArcGIS (v.10.8). *SSA* Sub-Saharan Africa

To determine the hot and cold areas of 115 regions throughout seven East African countries, Fig. 5 (a) and (c) displayed the findings of Getis-Ord Gi* statistics regarding the incidence of *P. falciparum* and *P. vivax* malaria, respectively. However, Anselins Local Moran's *I* findings for *P. falciparum* and *P. vivax*, respectively, were shown in Fig. 5 (b) and (d) to identify places with either a greater or lower incidence of malaria than the regions around them.

Due to the findings of our study, the incidence of *P. falciparum* malaria throughout the study period has placed all of South Sudan, Gambella, and the Southern Nations, Nationalities, and Peoples (SNNP) regions in Ethiopia and East Darfur in Sudan in hot spots. However, during the study period, the Northeast and Southeast parts of Kenya, all of Eritrea, Kassala in Sudan, Tigray, and the Afar region of Ethiopia are cold spots for the occurrence of *P. falciparum* malaria (Fig. 5 (a)).

The Gambella region in Ethiopia, Homa Bay, Siaya, Busia, Kakamega, and Vihiga regions in Kenya, as well as all of South Sudan, have higher rates of *P. falciparum* malaria than their bordering regions, as illustrated in Fig. 5 (b). However, compared to their neighbors, who have a high incidence of *P. falciparum* malaria, East Darfur in Sudan has a low incidence.

Based on the results of our study, the occurrence of *P. vivax* malaria in the study area caused all parts of Ethiopia-aside from the Tigray region, all regions of Djibouti, the Blue Nile, and Al Qadarif in Sudan, the Upper Nile in South Sudan, and Debubawi Keyih Bahri in Eritrea to become hot spots throughout the study period. Nonetheless, throughout the study period in seven East African countries, every region of Kenya was in a cold zone with the occurrence of *P. vivax* malaria (Fig. 5 (c)).

Based on this study, SNNP, Oromia, Harari, Dire Dawa, Afar, and Amhara in Ethiopia, Obock and Tadjourah in Djibouti, and Blue Nile in Sudan had higher rates of *P. vivax* malaria than their bordering regions. However, the incidence of *P. vivax* malaria is low in Addis Ababa, Ethiopia; the Upper Nile, South Sudan; Debubawi Keyih Bahri, Eritrea; Dikhil and Ali Sabieh, Djibouti; and Awdal and Woqoyi Galbeed, Somalia, while it is high in their neighbors during the study period (Fig. 5 (d)).

We provided the cluster and outlier analysis of the *P. falciparum* at regional level in Eastern Africa countries from 2011 to 2020 in supplemental file (Fig. S1), cluster and outlier analysis result of the *P. vivax* at regional level in Eastern Africa countries from 2011 to 2020 in supplemental file (Fig. S2), Hot Spot Analysis of the *P. falciparum* at regional level in Eastern Africa countries from 2011 to 2020 in supplemental file (Fig. S3), Hot Spot Analysis of the *P. vivax* at regional level in Eastern Africa countries from 2011 to 2020 in supplemental file (Fig. S4).

### Bayesian spatiotemporal model results

To determine the optimum model fit for estimating the incidence of *P. falciparum* and *P. vivax* malaria, we fitted and evaluated six spatiotemporal models with various distributions. While we employed Poisson, negative binomial, and zero-inflated Poisson distributions for *P. vivax* malaria incidence data, we offered Poisson and negative binomial distributions for *P. falciparum* malaria incidence. We fitted the *P. vivax* malaria incidence data with a zero-inflated Poisson distribution because the data contained zero values. To select the best model for *P. falciparum* and *P. vivax* malaria incidence, we fitted a parametric trend (Model par), a non-parametric dynamic trend (Model ST), two unstructured effects interacting ${v}_{i} and {\phi }_{t}$ (Model int I), the structured temporal main effect ${\gamma }_{t}$ and unstructured spatial effect ${v}_{i}$ (Model int II), the unstructured temporal effect ${\phi }_{t}$ and the spatial main effect ${u}_{i}$ (Model III), and the spatial and temporally structured effects interacting ${u}_{i}$ and ${\gamma }_{t}$ (Model int IV).

The results of this study revealed that the Poisson distribution was chosen for both *P. falciparum* and *P. vivax* malaria incidence data models. Furthermore, the results suggest that the spatial and temporally structured effect interact (Model int IV) was the best model fit for *P. falciparum* malaria incidence, with a DIC of 16,518.10, the lowest DIC among the models tested. However, the structured temporal main effect and unstructured spatial effect (Model int II) was the best model pit for *P. vivax* malaria incidence with a DIC of 6889.11 which is the smallest compared to other models (Table 1).

**Table 1:** Model comparisons of *Plasmodium falciparum* and *P. vivax* malaria incidence

Model	*P. falciparum*		*P. vivax*		
	Poisson	Negative Binomial	Poisson	Negative Binomial	Zero-inflated Poisson
	DIC	DIC	DIC	DIC	DIC
Model Par	219,782.72	24,993.69	195,309.46	12,420.54	163,737.70
Model ST	234,491.28	25,977.12	287,222.96	11,422.22	343,151.57
Model int I	16,596.10	24,785.34	7137.96	11,059.03	8959.45
Model int II*	16,567.14	23,195.17	**6889.11**	10,797.21	8405.86
Model int III	16,560.25	25,908.88	7085.47	11,170.08	8834.09
Model int IV**	**16,518.10**	18,254.96	7772.85	10,901.31	9267.17

^**^indicates the best model fit for *P. falciparum* and *for *P. vivax*; and the bold value indicates the selected distribution for the model of *P. falciparum* and *P. vivax* malaria incidence. *DIC* Deviance information criterion

In this study, the R-INLA result for both model fit: Model II for *P. vivax* and Model IV for *P. falciparum* reveal that the parameters estimated by INLA are ${{\varvec{\uptheta}}} = \left\{ {{\upalpha },{ }{{\varvec{\upxi}}}, {\varvec{u}},\user2{ \gamma },\phi ,{\varvec{\delta}}} \right\}$ with hyper-parameters $\uppsi =\left\{{\tau }_{u}, {\tau }_{v}, {\tau }_{\gamma }, {\tau }_{\phi }, {\tau }_{\delta }\right\}$.

For parameter estimation, α is the mean of *P. falciparum* or *P. vivax* MIR across 115 regions in 7 East African countries of SSA,$\xi$ is the area-specific residual, ${\varvec{u}}$ is the spatially structured residuals, $\gamma$ is the temporally structured effect, ${\varvec{\phi}}$ is the temporally unstructured effect, and $\delta$ is the spatially and temporally structured effects interaction for *P. falciparum* or temporal structured effect and unstructured spatial effects interaction for *P. vivax*.

Table 2 shows the results of the posterior mean and 95% credible interval for the intercept $\alpha$ and hyper-parameter for *P. falciparum* and *P. vivax* malaria incidence in 115 regions across the study period. The results of this study reveal that the intercept $\left( {\alpha \, = \, - 0.662} \right)$ of *P. falciparum* malaria incidence is statistically significant with credibility interval of (−0.679, −0.564), however, the *P. vivax* malaria incidence intercept $\left( {\alpha \, = 0.320} \right)$ with credibility interval of (−0.446, 1.085) is not statistically significant since the credibility interval does not include zero. In the present research, the posterior mean of the exponentiated intercept $\alpha$ for *P. falciparum* indicates a 46.31% reduction in MIR across 7 East African nations, with a 95% confidence interval ranging from 43.07% to 49.29% across the study period.

**Table 2:** Posterior mean and 95% credibility interval for the parameters and hyper-parameters of Poisson for *Plasmodium falciparum* and *P. vivax*

Parameter	*P. falciparum*	*P. vivax*
	Coeff (95% CrI)	Coeff (95% CrI)
Fixed effect		
Intercept (${\varvec{\upalpha}}$)	−0.622 (−0.679, −0.564)	0.320 (−0.446, 1.085)
Precision of hyper-parameters random effects		
BYM Spatial (${{\varvec{\tau}}}_{{\varvec{u}}}$)	2223.68 (146.65, 8828.27)	2202.94 (150.34, 8444.02)
BYM IID (${{\varvec{\tau}}}_{{\varvec{v}}}$**)**	1102.09 (73.64, 4304.15)	1113.06 (74.89, 4372.10)
Temporal random effect (${{\varvec{\tau}}}_{{\varvec{\gamma}}}$)	952.77 (91.33, 3729.64)	27.09 (1.60, 140.58)
Unstructured temporal effect (${{\varvec{\tau}}}_{{\varvec{\phi}}}$)	165.81 (49.68, 405.85)	13.29 (2.47, 40.22)
Space and time interaction (${{\varvec{\tau}}}_{{\varvec{\delta}}}$)	0.764 (0.689, 0.844)	0.151 (0.130, 0.174)

*Coeff*: coefficients, *CrI*: credible interval, *BYM*: Besag, York, and Mollie

We recall that the precision of hyper-parameters is specified as${\uptau } = 1/{\upsigma }^{2}$, whereas the variance is $\sigma^{2} = 1/\tau$. In this study, the spatial structured effect and spatial unstructured effect of the *P. falciparum* MIR exhibit low spatial variability within and between 115 regions, with posterior means of 2224.68 and 1102.09, respectively. Furthermore, the temporally structured effect for *P. falciparum* showed a low significant temporal variance of posterior mean of 952.77, temporal unstructured effect suggested a moderate temporal variation with a posterior mean of 165.81 across 115 regions. Moreover, the interaction combines spatial and temporal structured effects for *P. falciparum* MIR revealing that there is positive variability for each region, the parameter vector has an autoregressive structure on the time component, which is dependent on the ones of the other regions in 115 regions with a posterior mean of 0.764 over the study period (Table 2).

Besides, the study’s findings demonstrated that the spatially structured effect of *P. vivax MIR* has a low spatial variability, dependent on one of the other regions in 115 regions across the study period with a posterior mean of 2202.94. Additionally, the spatial unstructured effect of *P. vivax* MIR has a modest spatial variation and is independent of the other regions with a posterior mean of 1113.06. Moreover, the temporal structured effect of *P. vivax* MIR has a relatively low significant temporal variation on time component, with a posterior mean of 27.09 whereas, the temporal unstructured effect has a slightly significant temporal variance on time independent, with a posterior mean of 13.29 in 115 regions. Nevertheless, the combination of the structured temporal main effect and the unstructured spatial effect of *P. vivax* MIR has a substantial variance on the neighborhood structure specified using an RW in the 115 regions between 2011 and 2020, with a posterior mean of 0.151 (Table 2).

**Fig. 6:**
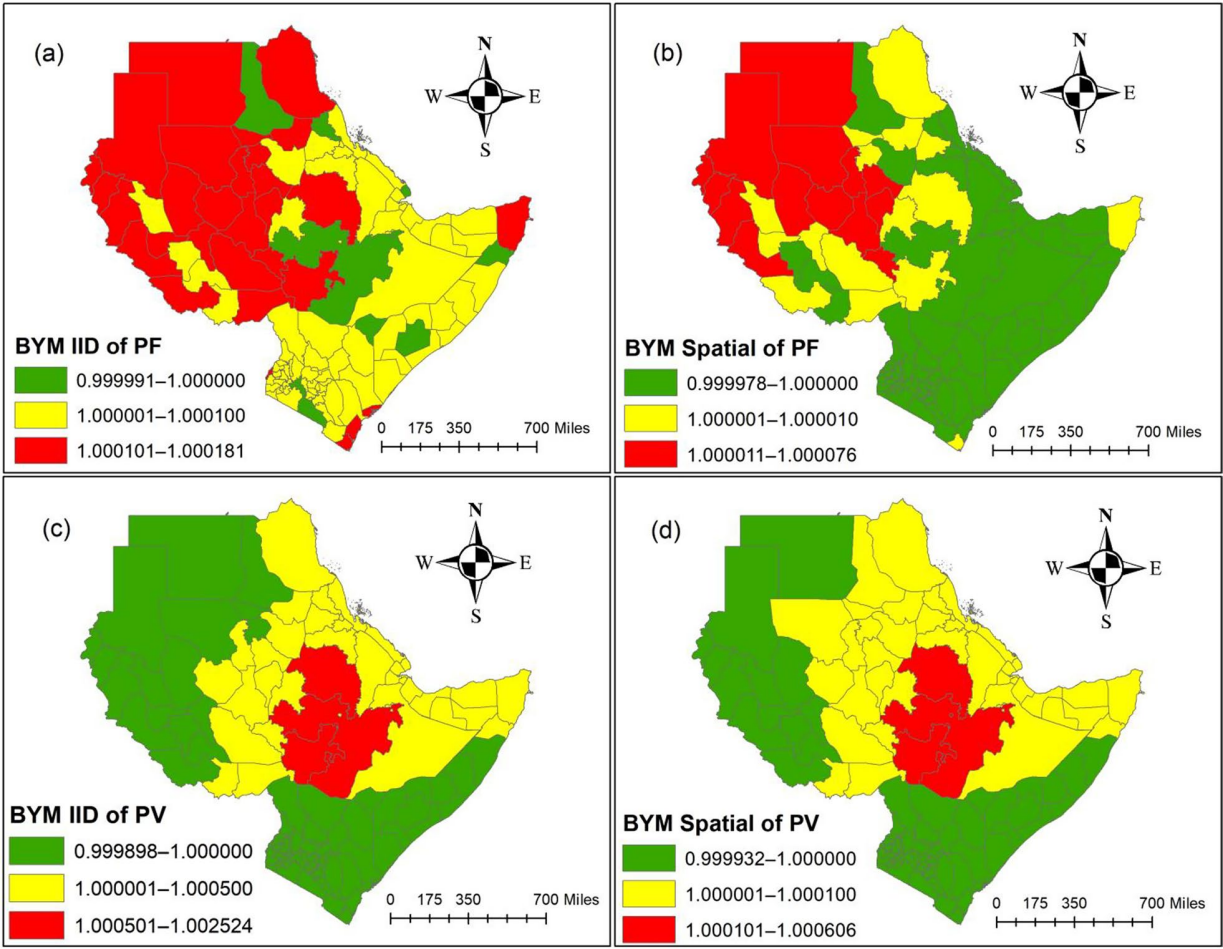
Spatial distribution of the posterior means of area-specific effects $\text{exp}({\upxi }_{\text{i}})$ and spatial structured effects $\text{exp}({u}_{\text{i}})$ from models in the Eastern Africa region, SSA, 2011 − 2020. **a** area-specific effects and (**b**) spatially random effects of *Plasmodium falciparum* (PF). **c** area-specific effects and (**d**) spatially random effects of *Plasmodium vivax* (PV). Source of shapefile: Database of Global Administrative Areas v.4.1 (www.gadm.org), own map output from ArcGIS (v.10.8). *BYM* Besag, York, and Mollie

Figure 6 (a) and (b) show the results of the relative risk of the area-specific and spatial structured effects for *P. falciparum* MIR, respectively, whereas Figs. 5 (c) and 6 (d) show the findings of the relative risk of each region specific and spatial structured effects for *P. vivax* MIR in 115 regions in 7 East African countries of SSA over the study period (2011–2020). The data shows that the MIR impacts of *P. falciparum* and *P. vivax* were lowest in certain areas and across 115 regions during the study period. In this study, the relative risk of *P. falciparum* for each region across the study period was higher in various areas of South Sudan, Sudan, Ethiopia, Kenya, and the Bari region of Somalia. In contrast, the relative risk of *P. falciparum* MIR for specific regions was lowest in River Nile in Sudan, Semenawi Keyih Bahri in Eritrea, Obock in Djibouti, Oromia and Harari in Ethiopia, Nugaal and Bay in Somalia, Makueni, Madera, and Taita Taveta in Kenya (Fig. 6 (a)). However, the relative risk of *P. falciparum* MIR for spatial structured effects was higher in the following regions: North, North Darfur, North Kurdufan, West Darfur, Central Darfur, South Darfur, East Darfur, West Kurdufan, South Kurdurfan, White Nile and Sennar in Sudan, West Equatoria and Upper Nile in South Sudan, and Gambella in Ethiopia (Fig. 6 (b)). The findings of this study demonstrated that the relative risk of *P. vivax* MIR in both specific regions and across regions was highest in the Amhara, Oromia, and SSNP regions of Ethiopia during the study period. However, the relative risk of *P. vivax* MIR is lowest in regions such as South Sudan, Kenya, Northwest and Southwest Sudan, and Central and Southern Somalia (Fig. 6. (c) and (d)).

**Fig. 7:**
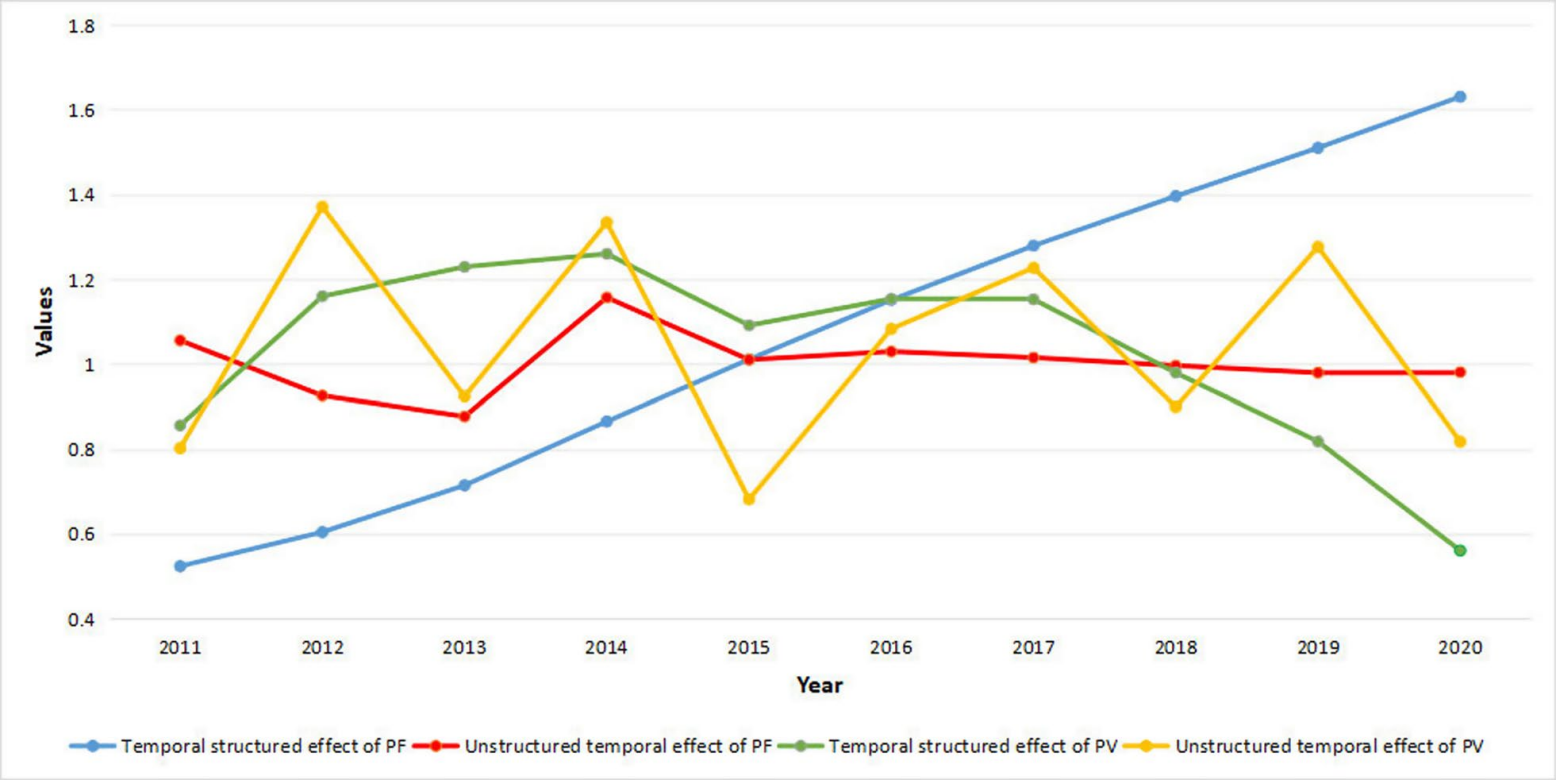
Posterior mean of temporal trends for *Plasmodium falciparum* (PF) and *Plasmodium vivax* (PV) malaria incidence: Temporal structured effect $\text{exp}({\upgamma }_{\text{t}})$ and unstructured temporal effect $\text{exp}({\upphi }_{\text{t}})$ in the study area from 2011 to 2020

Figure 7 depicted the relative risk results of temporal trends for temporal structured effect and temporal unstructured effect using an RW and independent identically distributed normal random effect specifications, respectively, for both *P. falciparum* and *P. vivax* MIR between 2011 and 2020 in the study area. In this study, the temporal structured effect of *P. falciparum* malaria relative risk (blue) is shown as linear increases through RW across 115 regions from 2011 to 2020. The temporal unstructured effect of *P. falciparum* malaria relative risk (red) shows falls from 2011 to 2013 and increases in 2014. Nevertheless, it suggests a uniform relative risk between 2015 and 2020 for time independence across 115 regions. Furthermore, the malaria relative risk for the temporal structured effect of *P. vivax* (green) indicates that the malaria relative risk increased from 2011 to 2014 before decreasing in 2015. However, it increased slightly between 2015 and 2017 and decreased from 2017 to 2020 via RW across 115 regions. Between 2011 and 2020, the temporal unstructured effect of *P. vivax* malaria relative risk (orange) jiggles more through time-independence across 115 regions.

**Fig. 8:**
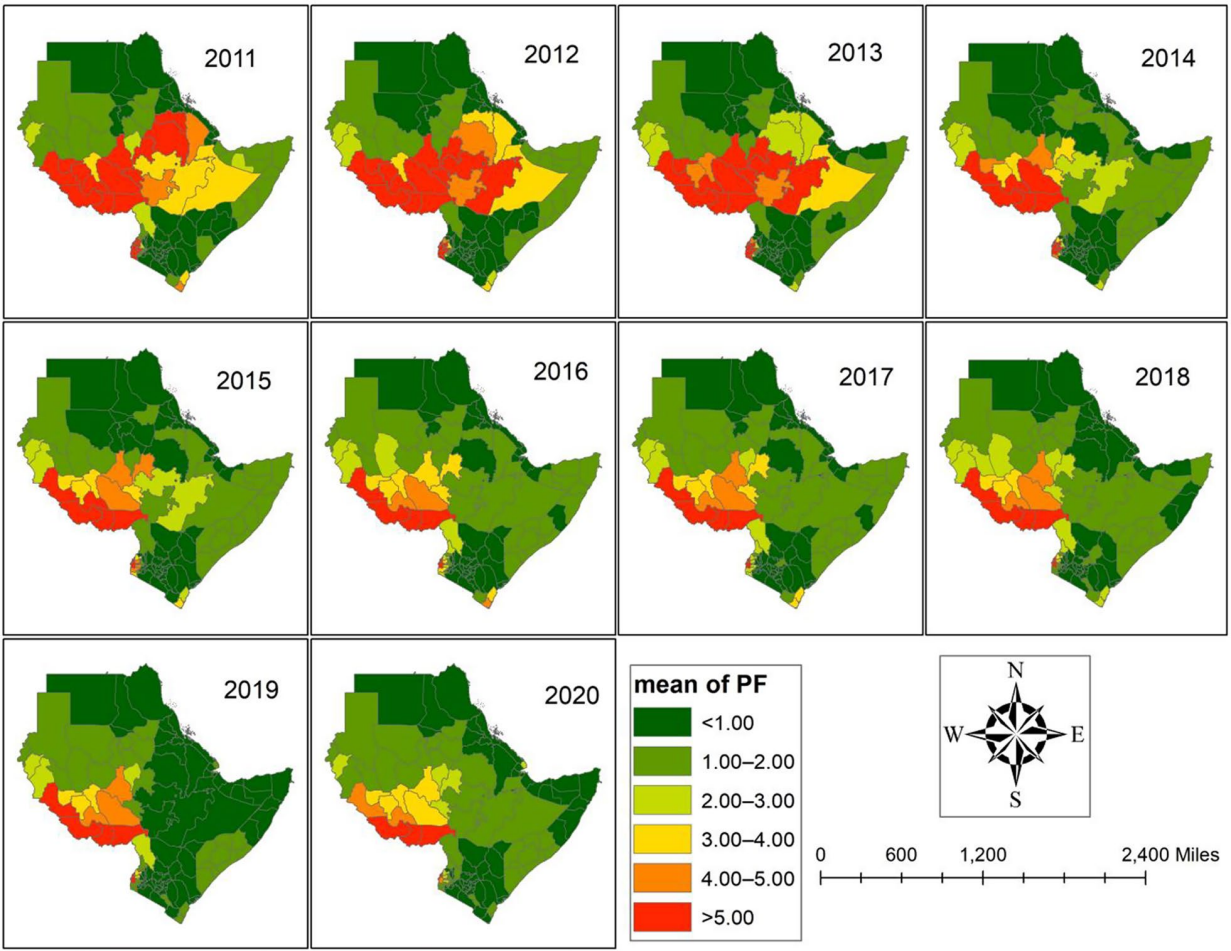
Posterior means of the spatial and temporal structured effect interact $\text{exp}({\updelta }_{\text{i}})$ for *P. falciparum* malaria incidence of 115 regions from 2011 to 2020. Source of shapefile: Database of Global Administrative Areas v.4.1 (www.gadm.org), own map output from ArcGIS (v.10.8)

Figure 8 depicts the malaria relative risk of the regional and temporal structured effect interaction for *P. falciparum* malaria incidence in 115 regions across 7 East African nations of SSA from 2011 to 2020. In this study, ten regions in South Sudan have a higher malaria risk due to the geographical and temporal structured effect interaction than other regions in terms of *P. falciparum* malaria incidence between 2011 and 2020. Furthermore, the territories around Ethiopia had a high malaria risk for *P. falciparum* between 2011 and 2013, while it declined from 2014 to 2020. However, Gambella and Benishagul Gumuz in Ethiopia, as well as Bungoma, Busia, Kakamega, Nandi, Vihiga, Siaya, Kisumu, Homa Bay, Migori, Kilifia, and Kwale in Kenya, have the highest malaria risk of *P. falciparum* between 2011 and 2020 in the study area, due to the interaction of geographical and temporal structured effects.

**Fig. 9:**
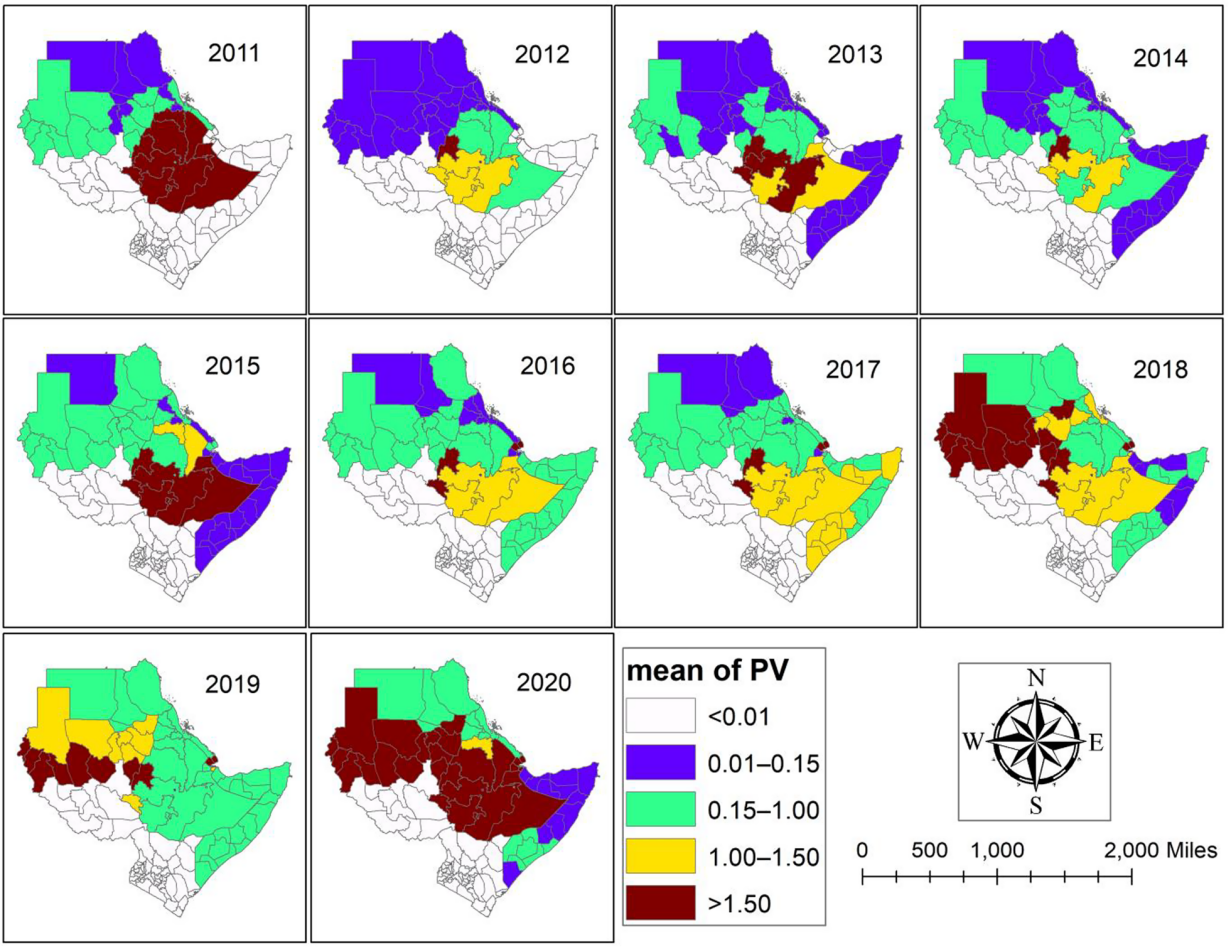
Posterior means of the combined temporal structured effect and unstructured spatial effect $\text{exp}({\updelta }_{\text{i}})$ for *P. vivax* malaria incidence of 115 regions from 2011 to 2020. Source of shapefile: Database of Global Administrative Areas v.4.1 (www.gadm.org), own map output from ArcGIS (v.10.8)

Figure 9 depicts the relative risk of the combined temporal structured effect and unstructured spatial effect for *P. vivax* malaria incidence in 115 regions between 2011 and 2020. The study's findings revealed that regions in Ethiopia had a high risk of *P. vivax* malaria during the study period. In 2011, all regions of Ethiopia except Addis Ababa had a high risk of *P. vivax* malaria in this study area. Furthermore, Benishagul Gumuz in Ethiopia had a higher malaria risk from *P. vivax* than other regions in 2012 and 2014. In 2013, Benishagul Gumuz, Gambella, and Oromia were at high risk of *P. vivax* malaria. In 2015, Benishagul Gumuz, Gambella, Oromia, SNNP, Somalia, Dire Dawa, and Harari in Ethiopia were at high risk of *P. vivax* malaria transmission. Furthermore, Obock and Tadjourah in Djibouti, as well as Benishagul Gumuz and Gambella in Ethiopia, are at high risk for *Plasmodium vivax* malaria in 2016 and 2017. Moreover, North Darfur, West Darfur, Central Darfur, South Darfur, East Darfur, North Kurdufan, West Kurdufan, South Kurdufan, White Nile, Sennar, Blue Nile, and Kassala in Sudan, Benishagul Gumuz and Gambella in Ethiopia, and Obock and Tadjourah in Djibouti are at high risk of *P. vivax* malaria in 2018. Nevertheless, West Darfur, Central Darfur, South Darfur, East Darfur, West Kurdufan, South Kurdufan, and Blue Nile in Sudan, Benishagul Gumuz in Ethiopia, and Obock and Tadjourah in Djibouti have a high *P. vivax* malaria risk in 2019. Finally, North Darfur, West Darfur, Central Darfur, South Darfur, East Darfur, North Kurdufan, West Kurdufan, South Kurdufan, White Nile, Sennar, Blue Nile, Al Jazirah, Al Qadarif, and Kassala in Sudan, Amhara, Afar, Oromia, Somalia, SNNP, Benishagul Gumuz, Gambella, and Dire Dawa in Ethiopia, Obock, Ali Sabieh, Dikhil, and Tadjourah in Djibouti, have a high malaria risk of *P. vivax* in 2020.

## Discussion

The regions surrounding South Sudan had the highest MIR observed value of *P. falciparum* in the present study. Additionally, there are some regions with a high MIR in West Ethiopia, West Kenya, and Southwest Ethiopia. Furthermore, the regions surrounding Sudan and Somalia show an increase during the study period. However, in several Ethiopian locations, the observed value of *P. falciparum* MIR declined between 2015 and 2019. In this study, no *P. vivax* malaria cases were reported in any regions around South Sudan and Kenya during the research period. Between 2011 and 2017, the highest number of *P. vivax* MIR per 1000 people was found in the regions surrounding Ethiopia. Apart from that, the Obock and Tadjourah regions of Djibouti have higher MIR from *P. vivax* between 2017 and 2020 than other regions in the research area. According to [11], *P. vivax* is endemic and consistently causes major clinical illness in the Horn of Africa (Ethiopia, Djibouti, Eritrea, and Somalia), South Sudan, and the island of Madagascar. This study found that between 2011 and 2020, the MIR detected values of *P. falciparum* and *P. vivax* fluctuated randomly in each study area. *P. vivax* MIR levels in the regions surrounding Ethiopia declined between 2012 and 2020. Nonetheless, Obock and Tadjourah in Djibouti have shown the higher increases in *P. vivax* MIR between 2015 and 2020. In 2017, it was discovered that levels of *P. vivax* have somewhat increased in different parts of Somalia. Furthermore, the *P. vivax* MIR remains constant during the study period in the regions surrounding Kenya, South Sudan, and Eritrea.

This study indicates that all of South Sudan's areas, Gambella, and SNNP regions in Ethiopia, as well as East Darfur in Sudan, are hotspots for *P. falciparum* malaria incidence across the study period. However, the Afar area of Ethiopia, Kassala in Sudan, Tigray, the northeast and southeast regions of Kenya, and all of Eritrea are cold places for *P. falciparum* malaria. Besides, the Gambella region in Ethiopia, Homa Bay, Siaya, Busia, Kakamega, and Vihiga regions in East Kenya, as well as all of South Sudan regions, have higher rates of *P. falciparum* malaria than their bordering regions. Nevertheless, East Darfur in Sudan has a low incidence compared to its neighbors, who have a high incidence of *P. falciparum* malaria. Based on the results of our study, the occurrence of *P. vivax* malaria in the study area caused all parts of Ethiopia-aside from the Tigray region, all regions of Djibouti, the Blue Nile, and Al Qadarif in Sudan, the Upper Nile in South Sudan, and Debubawi Keyih Bahri in Eritrea to become hot spots throughout the study period. Nonetheless, throughout the study period in seven East African countries, every region of Kenya within a cold zone of *P. vivax* malaria. However, *P. vivax* is the most common species outside of Africa [8] and shares the biggest burden in a few Sub-Saharan African (SSA) nations, including Ethiopia [9]. According to Gwitira et al. (2020), strategies to reduce or control malaria depend on objective and accurate characterization of its clusters as a first step towards identifying areas with elevated malaria risk for intervention prioritization, since malaria occurrence exhibits spatial heterogeneity [41].

Based on this study, SNNP, Oromia, Harari, Dire Dawa, Afar, and Amhara in Ethiopia, Obock and Tadjourah in Djibouti, and Blue Nile in Sudan had higher rates of *P. vivax* malaria than their bordering regions. However, the incidence of *P. vivax* malaria is low in Addis Ababa, Ethiopia; the Upper Nile, South Sudan; Debubawi Keyih Bahri, Eritrea; Dikhil and Ali Sabieh, Djibouti; and Awdal and Woqoyi Galbeed, Somalia, while it is high in their neighbors during the study period.

The Poisson distribution was used for the *P. falciparum* and *P. vivax* malaria incidence data models, according to the study's findings based on model comparison. Furthermore, the results suggest that the spatial and temporally structured effect interact model was the best model fit for *P. falciparum* malaria incidence. However, the structured temporal main effect and unstructured spatial effect model were the best model pit for *P. vivax* malaria incidence.

According to the current research, the posterior mean of the exponentiated intercept for *P. falciparum* reveals a 46.31% decrease in the MIR across seven East African countries, with a 95% confidence interval that extend from 43.07% to 49.29% across the study period between 2011 and 2020. The decrease in malaria incidence and mortality is primarily due to malaria control methods such as indoor residual spraying (IRS) and the usage of insecticide-treated nets [20]. As malaria transmission declines, preventative and control strategies will increasingly rely on precise understanding of the geographical distribution of high-risk geographic areas to aid in malaria elimination [42]. In this study, the spatial structured effect and spatial unstructured effect of the *P. falciparum* MIR exhibits low spatial variability within and between 115 regions. Furthermore, the temporally structured effect for *P. falciparum* showed a low significant temporal variance, but the temporal unstructured effect suggested a moderate temporal variation across 115 regions. Moreover, the interaction combines spatial and temporal structured effects for *P. falciparum* MIR revealing that there is positive variability for each region, the parameter vector has an autoregressive structure on the time component, which is dependent on the ones of the other regions in 115 regions over the study period.

Besides, the study's findings demonstrated that the spatially structured effect of *P. vivax* MIR has a low spatial variability, dependent on one of the other regions in 115 regions across the study period. Additionally, the spatial unstructured effect of *P. vivax* MIR has modest spatial variation and is independent of the other regions. Moreover, the temporal structured effect of *P. vivax* MIR has a relatively low significant temporal variation in time-component whereas the temporal unstructured effect has a slightly significant temporal variance on time independent across 115 regions. Nevertheless, the combination of the structured temporal main effect and the unstructured spatial effect of *P. vivax* MIR has a substantial variance in the neighborhood structure specified using a random walk in the 115 regions between 2011 and 2020. According to [43], there are still sporadic outbreaks of malaria that show spatial heterogeneity across various regions throughout time and space, even though the disease's burden is decreasing. It is crucial to map the spatial heterogeneity of malaria in order to comprehend the mechanisms of transmission.

The relative risk of *P. falciparum* for each region across the study period was higher in various areas of South Sudan, Sudan, Ethiopia, Kenya, and the Bari region of Somalia. In contrast, the relative risk of *P. falciparum* MIR for specific regions was lowest in River Nile in Sudan, Semenawi Keyih Bahri in Eritrea, Obock in Djibouti, Oromia and Harari in Ethiopia, Nugaal and Bay in Somalia, Makueni, Madera, and Taita Taveta in Kenya. However, the relative risk of *P. falciparum* MIR for spatial structured effects was higher in the following regions: North, North Darfur, North Kurdufan, West Darfur, Central Darfur, South Darfur, East Darfur, West Kurdufan, South Kurdurfan, White Nile and Sennar in Sudan, West Equatoria and Upper Nile in South Sudan, and Gambella in Ethiopia. The ecological settings and geographic ranges of the parasite species that can infect humans differ. A region may have a dominating species or all of them [2, 6, 7]. According to this study, both specific and across regions the relative risk of *P. vivax* MIR was highest in the Amhara, Oromia, and SSNP regions of Ethiopia during the study period. However, the relative risk of *P. vivax* MIR is lowest in regions such as South Sudan, Kenya, Northwest and Southwest Sudan, and Central and Southern Somalia.

In this study, the temporal structured effect of *P. falciparum* malaria relative risk is shown as linear increases through Random Walk across 115 regions from 2011 to 2020. The temporal unstructured effect of *P. falciparum* malaria relative risk shows falling from 2011 to 2013 and increases in 2014. It suggests a uniform relative risk between 2015 and 2020 for time independence across 115 regions. Furthermore, the malaria relative risk for the temporal structured effect of *P. vivax* indicates that the malaria relative risk increased from 2011 to 2014 before decreasing in 2015. However, it increased slightly between 2015 and 2017 and decreased from 2017 to 2020 via Random Walk across 115 regions. Between 2011 and 2020, the temporal unstructured effect of *P. vivax* malaria relative risk jiggles more through a time-independent across 115 regions.

Ten regions in South Sudan and some regions of West Kenya have a higher malaria risk due to the geographical and temporal structured effect interaction than other regions in terms of *P. falciparum* malaria incidence between 2011 and 2020. Furthermore, the territories around Ethiopia had a high malaria risk for *P. falciparum* between 2011 and 2013, while it declined from 2014 to 2020. This study indicates that between 2011 and 2018, as well as in 2020, there are a significant risk of *P. vivax* malaria in various Ethiopian regions. Furthermore, Obock and Tadjourah experienced high risk of *P. vivax* between 2016 and 2020 due to the structured temporal main effect and unstructured spatial effect. Nevertheless, from 2018 to 2020 many areas in Sudan were a high risk of *P. vivax* malaria. According to [44], malaria’s regional variation is mostly attributable to differences in environmental risk factors at both the macro (temperature, precipitation) and micro (local elevation, land use) spatial scales.

The limitations of this study should be considered when reviewing the results. First, we excluded the various socioeconomic and environmental factors that influence the risks of malaria incidence. Second, we did not include the coverage of malaria vector control intervention elements, which are crucial in identifying the populations in the research area that may be less protected. Lastly, the populations of the study area were projected, which might have resulted in an overestimation or an underestimation. To identify the populations in the research area that may be less protected, future researchers will use different models such as the Generalized Linear Model, Multiple Linear Regression Models, Logistic Regression, Survival Data Analysis, and Bayesian setting using Markov-chain Monte-Carlo simulation. These models will combine various socioeconomic and environmental factors that influence the risks of malaria incidence and the coverage of malaria vector control intervention elements. This will give health sectors solid evidence to eradicate malaria in high-transmission malaria regions.

## Conclusions

According to our study, the best spatiotemporal model fit for *P. falciparum* malaria incidence data is the spatially and temporally structured effect interact; for *P. vivax* malaria incidence data were the combination of the structured temporal main effect and unstructured spatial effect.

Throughout the study period, there was little variation in the spatially structured and spatially unstructured effects of *P. falciparum* malaria incidence within and between regions. Each region has a high variance in *P. falciparum* malaria incidence due to the interaction of geographical and temporal structured effects, dependent on each other through RW. Additionally, there is little variation in the spatially structured and unstructured spatial effects of *P. vivax* malaria incidence, which are dependent and independent of those of the other regions, respectively. The *P. vivax* malaria incidence varies greatly depending on the neighborhood structure specified by an RW in the regions between 2011 and 2020 when the structured temporal main effect and unstructured spatial effect are combined.

According to this study, the areas with the highest risk of *P. falciparum* malaria were Homa Bay, Siaya, Busia, Kakamega, and Vihita in Kenya; Gambella in Ethiopia; and all South Sudan regions. However, SNNP, Oromia, Harari, Afar, and Amhara in Ethiopia, as well as the Blue Nile in Sudan, had the highest risk of *P. vivax* malaria.

As a result, we recommend that spatiotemporal models be used to estimate a regional level trend while taking spatial-time interaction effects into account to identify locations that require immediate prevention and control interventions. Additionally, the global malaria control and eradication effort should focus especially on the South Sudan and Ethiopia regions to provide more intervention control to reduce the risk of *P. falciparum* and *P. vivax* malaria incidence, respectively. Finally, the malaria prevention, control, and eradication program could target locations with lower or higher malaria rates to eradicate malaria in East African countries.

## Supplementary Information

Supplementary file 1

## Data Availability

The malaria data used to support the outcome of this paper are available at the Malaria Atlas website at https://malariaatlas.org/.

## Abbreviations

BYM: Besag, York, and Mollie
CAR: Conditional autoregressive
DIC: Deviance information criterion
INLA: Integrated nested Laplace approximation
MAP: Malaria Atlas project
MIR: Malaria incidence rate
RW: Random walk
SNNP: Southern nations, nationalities, and peoples
SSA: Sub-Saharan Africa
