# Supplementary material for: Trend of malaria parasites infection in Ethiopia along an international border: a Bayesian spatio-temporal study
Source: Infect Dis Poverty. 2025 Jul 11;14:66. doi: 10.1186/s40249-025-01320-w (PMC12247342; doi:10.1186/s40249-025-01320-w)
Supplement: Supplementary file 1 [file 40249_2025_1320_MOESM1_ESM.docx]

**Supplemental files**

**Text S1**. Spatial Autocorrelation Statistics

**Global Moran’s I value**

One often used technique for evaluating spatial dependency in spatial lattice data is Moran's test [[1](#Ref1)]. This test can be directly linked to the data or to the residuals of a few spatial relapses. In this study, let $\{Z_{i(t)}:i=1,\ldots,m=115\}$ represent spatially referenced data (or residuals) for $m=115$ spatial locations (regions) at time $t$, where $t=2011, ..., 2020$. Then, we use the formula

| $I(t)=\frac{m\sum_{i=1}^{m} \sum_{j=1}^{m} w_{ij}(Z_{i(t)}-\bar{Z(t)})(Z_{j(t)}-\bar{Z(t)})}{(\sum_{i=1}^{m} \sum_{j=1}^{m} w_{ij}(\sum_{i=1}^{m} \left( Z_{i(t)}-\bar{Z(t)} \right)^{2})},$ | (S1) |
| --- | --- |

to Moran’s $I$ statistic, where $\bar{Z(t)}=(1/m)\sum_{i=1}^{m} Z_{i(t)}$ is the spatial mean at each specific year and $w_{ij}$ are spatial adjacency “weights” between locations $i$ and $j$ (where we require $w_{ii}=0$, for all $i=1,\ldots,115$). As a result, the formula for calculating the $z_{I(t)}-score$ for the Moran *I* statistic is $z_{I(t)}=\frac{I(t)-E(I(t))}{\sqrt{V[I(t)]}}$, where $E\left( I(t) \right)=\frac{-1}{(m-1)}$ and $V\left( I(t) \right)=E\left[ {I(t)}^{2} \right]-{(E[I(t)])}^{2}$, with $E(I(t))$ representing the expected value and $V(I(t))$ representing the variance.

Equation (**S1**) demonstrates that the Moran's I statistic is essentially a weighted form of the standard Pearson correlation coefficient, with the weights representing the geographic proximity weights. It takes values ranging from -1 to 1. If (**S1**) is positive, surrounding regions likely to have similar values, whereas if (**S1**) is negative, neighboring regions tend to have different values of *P. falciparum* or *P. vivax* malaria incidence in East African countries overall throughout the study period.

**Anselin’s Local Moran**

Luc Anselin (Anselin 1995) established the first LISA statistic, which is known as Anselin's Local Moran [[2](#Ref2)]. The method uses Moran's "I" statistics to specific regions, allowing them to be classified as comparable or dissimilar from the nearby pattern. The definition of “$I_{i(t)}$” is from [[3](#Ref3)]:

| $I_{i(t)}=\frac{(Z_{i(t)}-\bar{Z(t)})}{S_{Z(t)}^{2}}\sum_{j=1}^{m-1} \left[ w_{ij}(Z_{j(t)}-\bar{Z(t)}) \right]$ | (S2) |
| --- | --- |

where $t=2011, ..., 2020$, $Z_{i(t)}$ is the intensity of observation $i$ at time $t$, $\bar{Z}(t)$ is the mean intensity over all observations at time $t$, $Z_{j(t)}$ is intensity for all other observations, $j$ (where $j\neq i$) at time $t$, $S_{Z(t)}^{2}$ is the variance over all observations at time $t$, and $w_{ij}$ is a distance weight for the interaction between observations $i$ and $j$ during the study period. The first term in (S2) refers only to observation $i$ while the second term is the sum of the weighted values for all other observations (but not including $i$ itself). The expected “$I_{i(t)}$” is formulated as $E\left( I_{i(t)} \right)=\frac{\sum_{i=1}^{m} w_{ij}}{m-1},$ where $w_{ij}$ is the distance weight for the interaction between observations $i$ and $j$. The variance, standard deviation, and an approximate (pseudo) standardized score of $I_{i(t)}$ is $Var\left( I_{i(t)} \right)=\frac{\left( \sum_{i=1}^{m} \sum_{j=1}^{m} W_{ij}^{2} \right)\left( m-b_{2} \right)}{m-1}+\frac{2w_{i(kh)}\left( 2b_{2}-m \right)}{(m-1)(m-2)}+\frac{\left( \sum_{i=1}^{m} \sum_{j=1}^{m} W_{ij}^{2} \right)}{{(m-1)}^{2}}$, $S\left( I_{i(t)} \right)=\sqrt{Var(I_{i(t)})}$, and $Z\left( I_{i(t)} \right)=\frac{I_{i(t)}-E(I_{i(t)})}{S(I_{i(t)})}$. We define $b_{2}=\frac{\sum_{i=1}^{m} \frac{\left( Z_{i(t)}-\bar{Z(t)} \right)^{4}}{m}}{\left[ \sum_{i=1}^{m} \frac{\left( Z_{i(t)}-\bar{Z(t)} \right)^{4}}{m} \right]^{2}}$ and $2w_{i(kh)}=\sum_{k=1}^{m-1} \sum_{h=1}^{m-1} W_{ik}W_{ih}$, where $k\neq i$ and $h\neq i$. This term is twice the sum of the cross-products of all weights for $i$ with themselves, using $k$ and $h$ to maintain a strategic distance from the use of indistinguishable subscripts.

There are various applications for Anselin's Local Moran. It can, first, recognize areas that are distinct from those of its neighbors. This can serve as an excellent starting point for identifying regions that either have lower malaria incidence rates (a cold spot) or higher malaria incidence rates (a hot spot) than the neighboring regions. Researchers may focus on the problems that make the area more vulnerable to hot spots or cold spots to determine the factors that reduce or increase the incidence of malaria throughout the study period. Second, identifying "outliers," or areas that differ from their neighbors, is another use of Anselin's Local Moran statistic. In this case, regions with a high negative I esteem (e.g., with an “$I_{i(t)}$” smaller than two standard deviations underneath the mean) are demonstrative of outliers. They either have a high incidence rate relative to the low incidence rate of their neighbors, or they exhibit the opposite pattern, with low incidence rates among high incidence areas.

**Getis-Ord Local G***

The Getis-Ord Local G* statistic applies the Getis-Ord "G" statistic to individual regions to examine whether certain locations are spatially related to their neighbors. Unlike the global Getis-Ord G*, the Getis-Ord Local G* is applied to each region, much like Anselin's Local Moran. The formulation offered here is based on that of Wong and Lee (2005) [[4](#Ref4)]. In this study, the G*(t) value at time $t=2011, .., 2020$ is calculated concerning a specified search distance, namely:

| $G^{*}(t)=\frac{\sum_{i=1}^{m} \sum_{j=1}^{m} w_{i,j}x_{i}x_{j}}{\sum_{i=1}^{m} \sum_{j=1}^{m} x_{i}x_{j}}, \forall_{j\neq i}$ | (S3) |
| --- | --- |

where $w_{i,j}$ is the spatial weight between features $i$ and $j$, and $x_{i}$ and $x_{j}$ are the attribute values for features $i$ and $j$, $m$ is the number of features in the dataset (115 regions) and $\forall_{j\neq i}$ indicates that features $i$ and $j$ cannot be the same feature. Therefore, $z_{G^{*}(t)}=\frac{G^{*}(t)-E[G^{*}(t)]}{\sqrt{V[G^{*}(t)]}}$ yields the $z_{G^{*}(t)}-score$ for the statistic, where $V[G^{*}(t)]=E[{G^{*}(t)}^{2}]-(E{[G^{*}(t)])}^{2}$ and $E[G^{*}(t)]=\frac{\sum_{i=1}^{n} \sum_{j=1}^{n} w_{i,j}}{n(n-1)}, \forall_{j\neq i}$.

The Getis-Ord Local G* is excellent at distinguishing hot and cold locations. As previously demonstrated, Anselin's Local Moran can only detect positive or negative spatial autocorrelation, or how similar or unlike the regions are to one another. Those areas with positive spatial autocorrelation may be the result of high-value regions being close to another high-value region, or they may be the result of low-value regions being close to another low-value region of *P. falciparum* or *P. vivax* malaria incidence in East African nations between 2011 and 2020. The Getis-Ord Local G* is able to differentiate between the two types.

**References**

1. Waller LA, Gotway CA. Applied Spatial Statistics for Public Health Data. Hoboken, NJ, USA: John Wiley & Sons, Inc.; 2004.

2. Anselin L. Local Indicators of Spatial Association-LISA. Geogr Anal. 1995;27:93–115.

3. Getis A, Ord J. Local spatial statistics: an overview. In: Longley P, Batty M, editors. Spatial Analysis: Modelling in a GIS Environment. New York: John Wiley & Sons; 1996. p. 261–77.

4. Wong DWS, Lee J. Statistical analysis of geographic information with ArcView GIS and ArcGIS. Hoboken, N.J.: John Wiley & Sons; 2005.

**Table S1** Global Moran’s I autocorrelation value for annual malaria incidence cases of P*lasmodium falciparum* and *P.vivax* in the East Africa countries, 2011−2020

| **Year** | ***P. falciparum*** | | | | ***P. vivax*** | | | |
| --- | --- | --- | --- | --- | --- | --- | --- | --- |
|  | **Moran’s I** | **Variance** | **Z-score** | **P-value** | **Moran’s I** | **Variance** | **Z-score** | **P-value** |
| **2011** | 0.489689 | 0.001770 | 11.84711 | 0.001 | 0.330101 | 0.001713 | 8.18826 | 0.001 |
| **2012** | 0.505370 | 0.001784 | 12.17196 | 0.001 | 0.382063 | 0.001702 | 9.47248 | 0.001 |
| **2013** | 0.514200 | 0.001807 | 12.30381 | 0.001 | 0.395372 | 0.001659 | 9.92337 | 0.001 |
| **2014** | 0.501608 | 0.001801 | 12.02499 | 0.001 | 0.409395 | 0.001686 | 10.18368 | 0.001 |
| **2015** | 0.517609 | 0.001799 | 12.40971 | 0.001 | 0.401972 | 0.001708 | 9.93723 | 0.001 |
| **2016** | 0.496624 | 0.001797 | 11.92337 | 0.001 | 0.339546 | 0.001729 | 8.37707 | 0.001 |
| **2017** | 0.537914 | 0.001788 | 12.92825 | 0.001 | 0.237993 | 0.001310 | 6.81792 | 0.001 |
| **2018** | 0.512486 | 0.001802 | 12.27989 | 0.001 | 0.211645 | 0.000996 | 6.98380 | 0.001 |
| **2019** | 0.431367 | 0.001737 | 10.55943 | 0.001 | 0.217911 | 0.001029 | 7.06811 | 0.001 |
| **2020** | 0.454563 | 0.001719 | 11.17513 | 0.001 | 0.211244 | 0.000960 | 7.09921 | 0.001 |


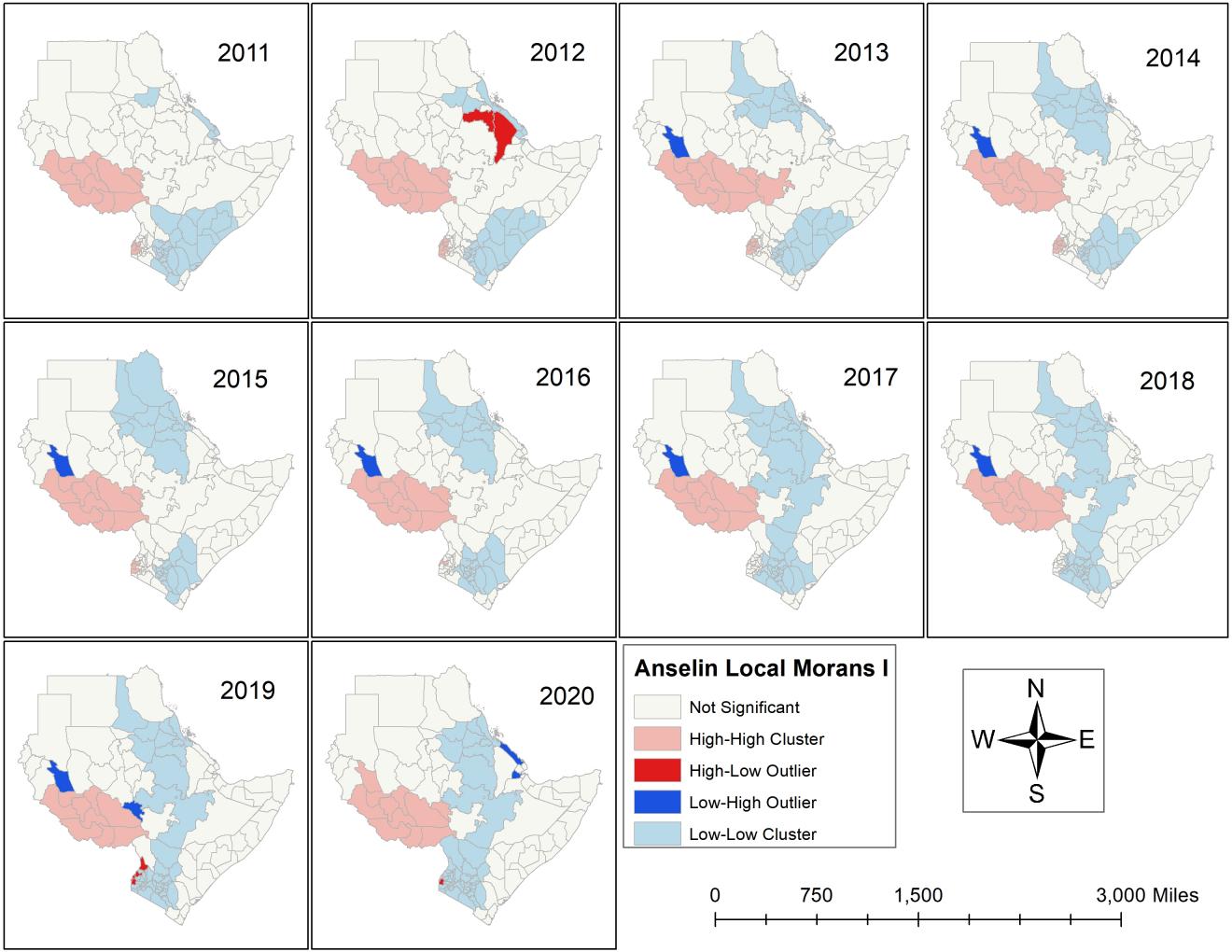


**Fig. S1** Cluster and outlier analysis of the P*lasmodium falciparum* at regional level in Eastern Africa countries from 2011 to 2020


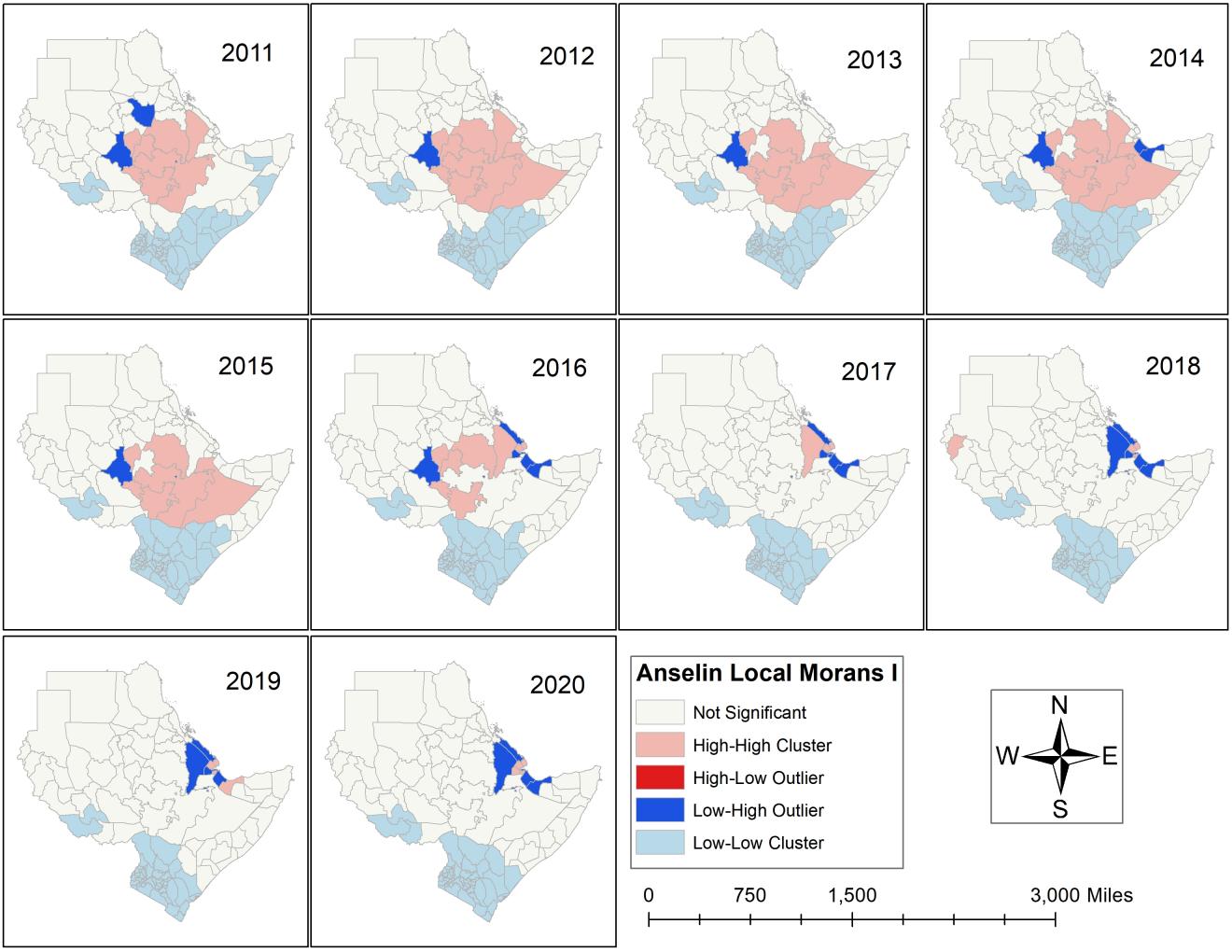


**Fig. S2** Cluster and outlier analysis of the P*lasmodium vivax* at regional level in Eastern Africa countries from 2011 to 2020


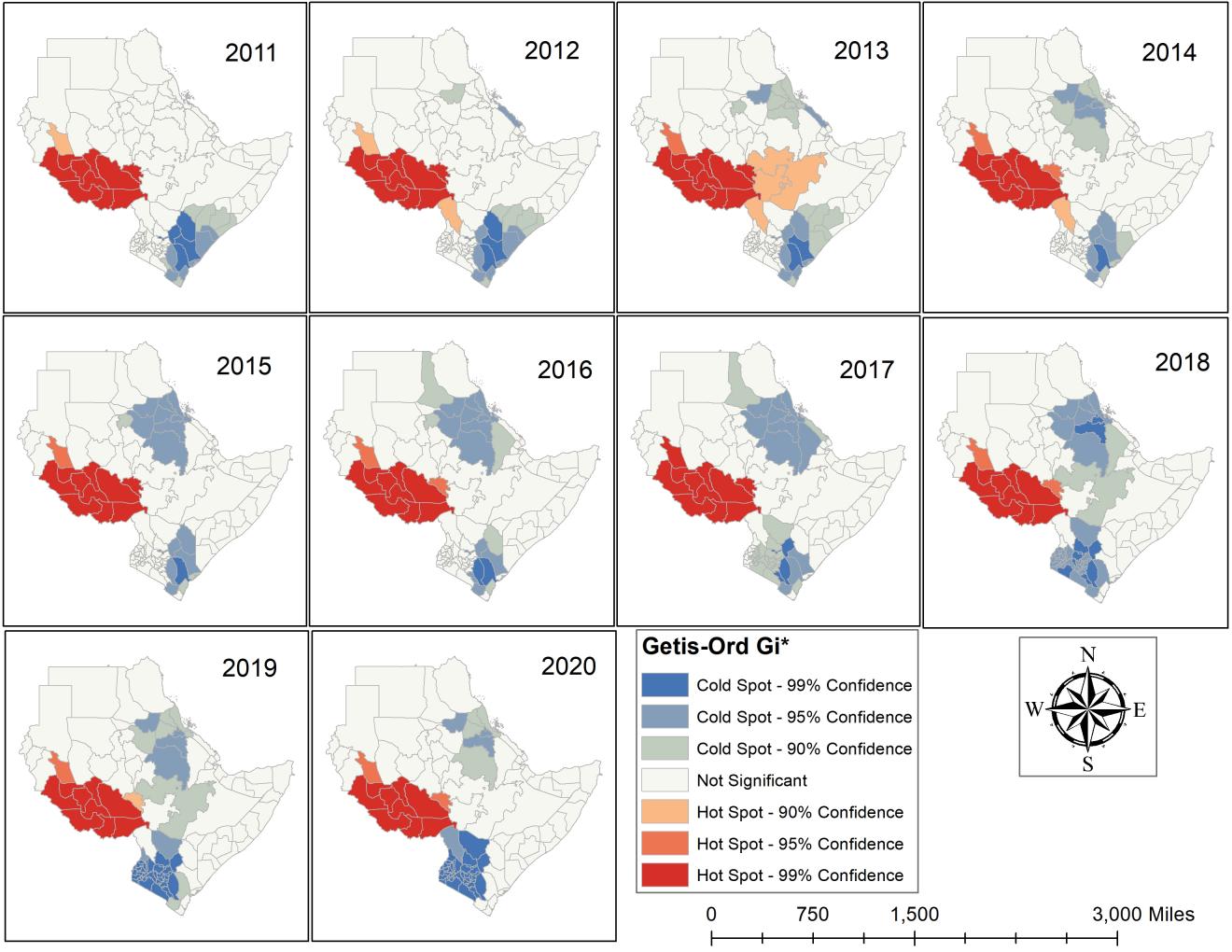


**Fig. S3** Hot Spot Analysis of the P*lasmodium falciparum* at regional level in Eastern Africa countries from 2011 to 2020


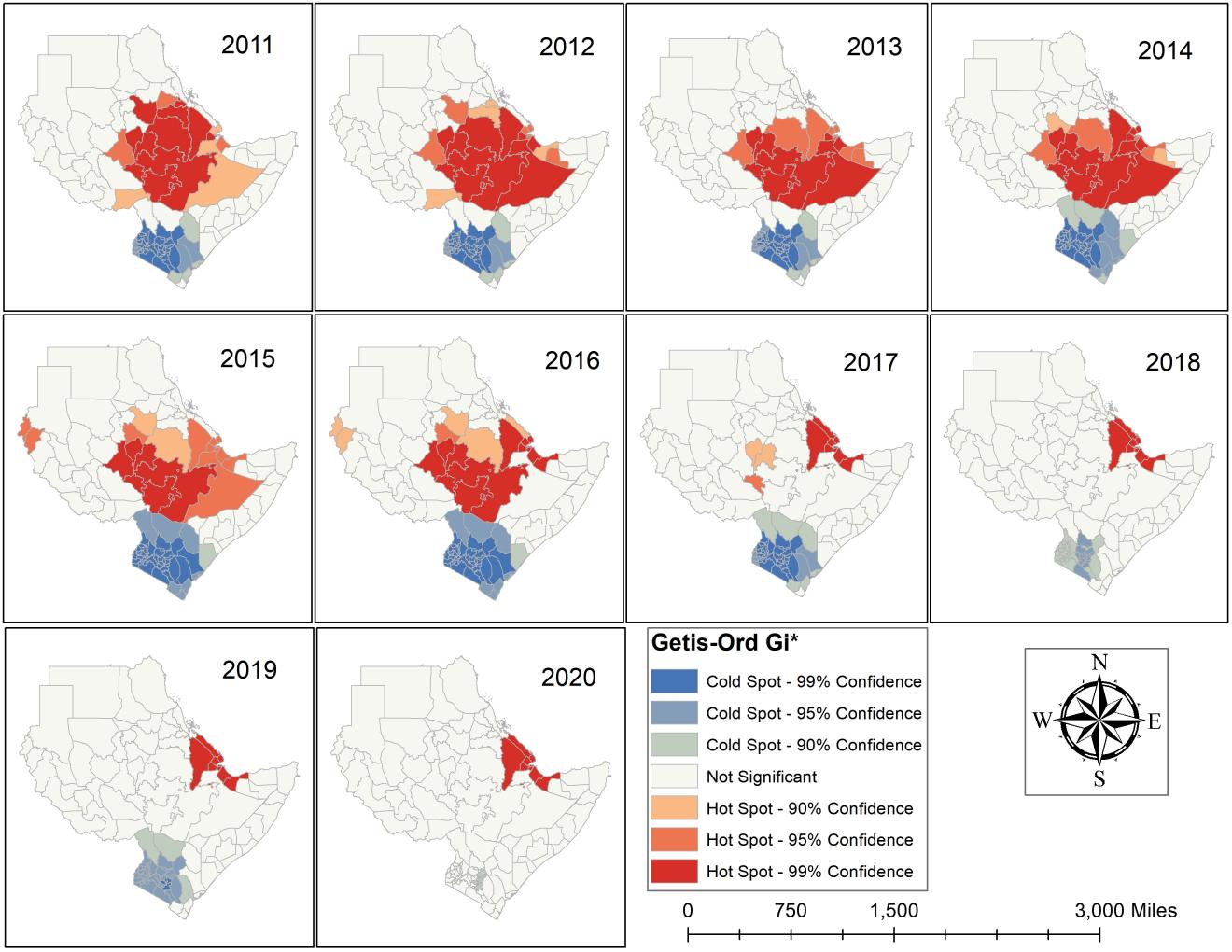


**Fig. S4** Hot Spot Analysis of the P*lasmodium vivax* at regional level in Eastern Africa countries from 2011 to 2020

**Table S2** Observed value of the *P. falciparum* yearly malaria incidence rates

| **RID** | **Country** | **Region** | ***P. falciparum* yearly malaria incidence rates** | | | | | | | | | |
| --- | --- | --- | --- | --- | --- | --- | --- | --- | --- | --- | --- | --- |
|  |  |  | **2011** | **2012** | **2013** | **2014** | **2015** | **2016** | **2017** | **2018** | **2019** | **2020** |
| 1 | Djibouti | Ali Sabieh | 0.029 | 0.019 | 1.104 | 8.650 | 7.837 | 9.838 | 8.183 | 12.394 | 25.797 | 28.244 |
| 2 | Djibouti | Dikhil | 0.018 | 0.011 | 0.776 | 5.891 | 5.368 | 5.977 | 4.123 | 6.671 | 17.543 | 19.054 |
| 3 | Djibouti | Djibouti | 0.001 | 0.001 | 0.031 | 0.292 | 0.276 | 0.400 | 0.323 | 0.537 | 1.049 | 1.204 |
| 4 | Djibouti | Obock | 0.988 | 0.498 | 25.562 | 195.540 | 175.264 | 225.279 | 181.640 | 293.475 | 564.791 | 577.906 |
| 5 | Djibouti | Tadjourah | 0.520 | 0.313 | 18.503 | 159.214 | 148.138 | 188.850 | 150.466 | 240.826 | 464.459 | 475.873 |
| 6 | Eritrea | Anseba | 10.817 | 5.218 | 3.068 | 7.432 | 5.204 | 8.445 | 9.995 | 9.760 | 21.318 | 18.493 |
| 7 | Eritrea | Debub | 16.876 | 7.219 | 3.590 | 7.961 | 5.419 | 9.430 | 11.888 | 11.929 | 27.247 | 23.523 |
| 8 | Eritrea | Debubawi Keyih Bahri | 20.039 | 11.383 | 8.051 | 21.167 | 14.368 | 22.729 | 27.981 | 26.938 | 49.283 | 38.858 |
| 9 | Eritrea | Gash Barka | 25.223 | 20.156 | 17.246 | 46.979 | 28.853 | 32.427 | 25.971 | 19.743 | 37.881 | 31.834 |
| 10 | Eritrea | Maekel | 8.508 | 3.645 | 1.914 | 4.530 | 3.061 | 5.078 | 5.952 | 5.747 | 12.641 | 11.188 |
| 11 | Eritrea | Semenawi Keyih Bahri | 12.619 | 5.400 | 2.935 | 7.030 | 5.050 | 8.902 | 11.386 | 11.494 | 25.331 | 21.942 |
| 12 | Ethiopia | Addis Abeba | 9.626 | 15.346 | 20.996 | 10.605 | 9.445 | 5.558 | 3.125 | 1.851 | 1.210 | 2.096 |
| 13 | Ethiopia | Afar | 103.034 | 83.537 | 73.561 | 32.471 | 31.047 | 26.445 | 28.201 | 28.456 | 24.955 | 47.345 |
| 14 | Ethiopia | Amhara | 170.748 | 94.370 | 55.129 | 19.485 | 16.871 | 15.363 | 18.462 | 19.544 | 17.970 | 35.671 |
| 15 | Ethiopia | Benshangul-Gumaz | 158.665 | 164.143 | 180.352 | 92.433 | 100.060 | 96.476 | 103.058 | 83.124 | 62.840 | 108.373 |
| 16 | Ethiopia | Dire Dawa | 111.106 | 161.693 | 207.417 | 102.640 | 91.223 | 53.527 | 33.617 | 21.022 | 15.560 | 34.157 |
| 17 | Ethiopia | Gambela Peoples | 157.130 | 170.130 | 185.380 | 97.120 | 109.692 | 111.700 | 129.791 | 110.600 | 81.550 | 129.663 |
| 18 | Ethiopia | Harari People | 105.712 | 153.463 | 194.802 | 93.083 | 79.083 | 41.391 | 22.781 | 14.031 | 10.808 | 25.305 |
| 19 | Ethiopia | Oromia | 79.098 | 145.622 | 212.199 | 109.411 | 109.465 | 78.336 | 53.684 | 35.482 | 25.100 | 45.196 |
| 20 | Ethiopia | Somali | 79.617 | 75.989 | 82.024 | 43.266 | 48.328 | 47.370 | 57.575 | 57.495 | 46.801 | 78.538 |
| 21 | Ethiopia | Southern Nations, Nationalities and Peoples | 112.149 | 115.495 | 123.224 | 60.760 | 62.178 | 52.077 | 51.407 | 44.520 | 35.464 | 59.471 |
| 22 | Ethiopia | Tigray | 136.260 | 75.251 | 44.366 | 16.732 | 16.094 | 18.689 | 28.559 | 32.819 | 27.861 | 44.951 |
| 23 | Kenya | Baringo | 12.848 | 16.579 | 23.560 | 26.825 | 27.341 | 27.968 | 28.962 | 32.537 | 37.334 | 40.041 |
| 24 | Kenya | Bomet | 7.318 | 8.443 | 9.936 | 10.872 | 11.565 | 11.472 | 11.878 | 14.063 | 18.153 | 22.518 |
| 25 | Kenya | Bungoma | 149.956 | 154.375 | 151.371 | 148.956 | 151.582 | 153.870 | 145.955 | 123.769 | 92.918 | 49.163 |
| 26 | Kenya | Busia | 370.526 | 338.746 | 298.963 | 273.741 | 269.342 | 279.227 | 272.407 | 235.897 | 169.331 | 81.657 |
| 27 | Kenya | Elgeyo-Marakwet | 6.668 | 8.008 | 10.900 | 12.537 | 13.631 | 16.315 | 19.149 | 21.328 | 22.416 | 19.374 |
| 28 | Kenya | Embu | 12.834 | 18.377 | 27.725 | 34.529 | 37.346 | 36.378 | 36.949 | 43.015 | 53.876 | 67.980 |
| 29 | Kenya | Garissa | 13.795 | 15.600 | 19.627 | 23.517 | 27.980 | 33.621 | 41.354 | 56.133 | 73.832 | 92.590 |
| 30 | Kenya | Homa Bay | 178.546 | 194.427 | 220.191 | 216.948 | 195.083 | 159.007 | 120.861 | 82.063 | 55.528 | 40.450 |
| 31 | Kenya | Isiolo | 12.380 | 17.893 | 28.751 | 35.771 | 38.250 | 39.976 | 41.495 | 49.493 | 58.696 | 69.295 |
| 32 | Kenya | Kajiado | 20.519 | 23.259 | 27.189 | 29.897 | 31.853 | 33.737 | 36.765 | 43.722 | 53.639 | 60.487 |
| 33 | Kenya | Kakamega | 193.916 | 207.657 | 207.477 | 195.733 | 178.420 | 152.783 | 123.311 | 96.285 | 73.402 | 43.100 |
| 34 | Kenya | Kericho | 26.236 | 33.327 | 38.895 | 38.822 | 33.496 | 23.868 | 17.463 | 16.299 | 18.800 | 22.079 |
| 35 | Kenya | Kiambu | 13.042 | 15.863 | 18.614 | 19.513 | 18.406 | 15.723 | 14.313 | 15.398 | 18.534 | 22.212 |
| 36 | Kenya | Kilifi | 70.789 | 64.695 | 62.122 | 66.117 | 78.053 | 98.980 | 118.227 | 134.424 | 144.289 | 140.920 |
| 37 | Kenya | Kirinyaga | 14.298 | 20.739 | 31.211 | 38.093 | 39.663 | 37.063 | 36.036 | 40.150 | 49.086 | 60.770 |
| 38 | Kenya | Kisii | 47.805 | 56.920 | 64.648 | 65.514 | 62.004 | 50.453 | 39.815 | 34.660 | 33.661 | 30.466 |
| 39 | Kenya | Kisumu | 220.011 | 246.157 | 258.666 | 239.966 | 198.231 | 138.105 | 86.293 | 60.481 | 46.800 | 37.038 |
| 40 | Kenya | Kitui | 14.954 | 18.032 | 23.808 | 26.582 | 27.173 | 29.220 | 32.923 | 42.698 | 59.570 | 80.613 |
| 41 | Kenya | Kwale | 109.308 | 113.824 | 124.344 | 131.792 | 137.053 | 143.067 | 140.607 | 139.619 | 136.572 | 126.184 |
| 42 | Kenya | Laikipia | 8.621 | 11.903 | 17.962 | 20.224 | 19.133 | 17.055 | 16.102 | 16.847 | 20.314 | 23.045 |
| 43 | Kenya | Lamu | 17.112 | 18.893 | 23.584 | 26.843 | 30.596 | 41.527 | 57.444 | 77.621 | 103.153 | 129.521 |
| 44 | Kenya | Machakos | 19.144 | 21.752 | 25.568 | 27.880 | 28.727 | 28.870 | 30.968 | 37.860 | 49.849 | 62.894 |
| 45 | Kenya | Makueni | 14.120 | 16.860 | 22.582 | 27.084 | 30.177 | 34.904 | 40.823 | 52.128 | 69.029 | 84.456 |
| 46 | Kenya | Mandera | 12.710 | 15.372 | 21.646 | 28.550 | 36.436 | 46.856 | 59.432 | 79.635 | 99.197 | 110.999 |
| 47 | Kenya | Marsabit | 18.255 | 20.115 | 25.246 | 30.287 | 36.048 | 43.534 | 52.466 | 65.426 | 74.327 | 78.845 |
| 48 | Kenya | Meru | 12.643 | 18.302 | 28.813 | 37.204 | 41.199 | 40.595 | 40.744 | 45.825 | 54.374 | 63.493 |
| 49 | Kenya | Migori | 134.709 | 149.322 | 163.051 | 160.901 | 149.612 | 123.262 | 91.886 | 65.143 | 49.139 | 38.413 |
| 50 | Kenya | Mombasa | 127.992 | 104.508 | 85.325 | 79.301 | 84.491 | 100.542 | 113.275 | 118.410 | 112.947 | 93.035 |
| 51 | Kenya | Murang'a | 17.057 | 23.165 | 31.450 | 36.286 | 35.886 | 31.431 | 29.033 | 31.772 | 38.931 | 48.362 |
| 52 | Kenya | Nairobi | 0.746 | 0.829 | 0.902 | 0.903 | 0.862 | 0.737 | 0.718 | 0.783 | 0.967 | 1.130 |
| 53 | Kenya | Nakuru | 11.202 | 14.760 | 19.324 | 21.051 | 20.075 | 16.410 | 13.864 | 13.820 | 15.876 | 17.854 |
| 54 | Kenya | Nandi | 102.027 | 126.655 | 142.038 | 135.266 | 109.195 | 67.948 | 39.534 | 28.086 | 24.100 | 21.015 |
| 55 | Kenya | Narok | 5.758 | 7.519 | 10.583 | 12.315 | 12.671 | 12.221 | 12.017 | 13.404 | 16.427 | 18.730 |
| 56 | Kenya | Nyamira | 32.515 | 38.611 | 42.728 | 42.049 | 38.705 | 31.422 | 26.288 | 25.322 | 28.075 | 30.087 |
| 57 | Kenya | Nyandarua | 0.171 | 0.251 | 0.330 | 0.327 | 0.275 | 0.202 | 0.166 | 0.167 | 0.197 | 0.234 |
| 58 | Kenya | Nyeri | 11.995 | 16.553 | 23.182 | 27.259 | 27.428 | 24.045 | 21.599 | 22.616 | 26.477 | 31.752 |
| 59 | Kenya | Samburu | 10.863 | 14.504 | 21.539 | 28.161 | 33.387 | 36.655 | 39.077 | 44.249 | 49.666 | 50.999 |
| 60 | Kenya | Siaya | 402.703 | 376.785 | 339.092 | 304.549 | 289.919 | 295.882 | 285.524 | 247.345 | 183.539 | 96.160 |
| 61 | Kenya | Taita Taveta | 23.330 | 27.238 | 35.846 | 41.519 | 45.074 | 51.754 | 58.087 | 68.343 | 81.656 | 89.660 |
| 62 | Kenya | Tana River | 15.765 | 20.192 | 30.025 | 36.154 | 40.374 | 45.914 | 52.948 | 66.022 | 85.473 | 110.913 |
| 63 | Kenya | Tharaka-Nithi | 14.017 | 19.822 | 29.460 | 36.388 | 39.603 | 38.851 | 39.508 | 45.652 | 55.493 | 68.291 |
| 64 | Kenya | Trans Nzoia | 28.490 | 27.844 | 28.344 | 32.069 | 40.835 | 57.984 | 75.231 | 78.417 | 68.741 | 39.997 |
| 65 | Kenya | Turkana | 56.904 | 54.548 | 55.188 | 61.279 | 77.713 | 108.976 | 133.313 | 136.033 | 102.055 | 52.411 |
| 66 | Kenya | Uasin Gishu | 13.993 | 16.722 | 19.712 | 21.899 | 23.560 | 24.084 | 25.055 | 25.015 | 24.748 | 18.803 |
| 67 | Kenya | Vihiga | 273.325 | 289.041 | 286.800 | 265.399 | 229.108 | 173.034 | 119.061 | 84.325 | 63.068 | 41.648 |
| 68 | Kenya | Wajir | 10.965 | 13.165 | 16.640 | 20.365 | 24.564 | 30.845 | 40.235 | 56.843 | 73.749 | 86.502 |
| 69 | Kenya | West Pokot | 30.396 | 33.796 | 45.096 | 58.443 | 77.976 | 116.177 | 148.139 | 150.577 | 120.731 | 60.513 |
| 70 | Somalia | Awdal | 31.915 | 30.462 | 30.776 | 32.573 | 35.857 | 41.870 | 51.728 | 59.411 | 64.426 | 69.832 |
| 71 | Somalia | Bakool | 22.198 | 25.235 | 35.249 | 48.420 | 67.191 | 93.382 | 123.716 | 150.329 | 154.857 | 152.198 |
| 72 | Somalia | Banaadir | 22.948 | 22.373 | 22.292 | 25.755 | 32.623 | 48.508 | 71.040 | 90.319 | 102.355 | 108.313 |
| 73 | Somalia | Bari | 39.572 | 34.879 | 34.638 | 39.757 | 47.879 | 63.952 | 86.781 | 102.687 | 102.581 | 97.528 |
| 74 | Somalia | Bay | 18.688 | 21.397 | 30.597 | 43.896 | 63.791 | 93.448 | 126.233 | 154.396 | 162.472 | 162.182 |
| 75 | Somalia | Galguduud | 30.644 | 27.005 | 26.930 | 28.835 | 32.564 | 41.482 | 55.128 | 67.652 | 69.654 | 69.444 |
| 76 | Somalia | Gedo | 16.508 | 19.721 | 28.162 | 40.717 | 58.923 | 86.712 | 117.817 | 144.780 | 148.644 | 143.366 |
| 77 | Somalia | Hiiraan | 37.429 | 35.438 | 38.192 | 43.164 | 52.317 | 68.838 | 88.538 | 106.770 | 108.726 | 108.442 |
| 78 | Somalia | Jubbada Dhexe | 25.752 | 29.858 | 41.019 | 56.236 | 75.122 | 103.523 | 136.439 | 161.084 | 165.217 | 156.806 |
| 79 | Somalia | Jubbada Hoose | 38.011 | 36.969 | 41.009 | 50.363 | 64.682 | 91.095 | 123.879 | 146.304 | 146.053 | 132.920 |
| 80 | Somalia | Mudug | 29.986 | 28.964 | 30.837 | 35.321 | 41.613 | 53.461 | 69.123 | 81.418 | 81.823 | 78.815 |
| 81 | Somalia | Nugaal | 34.097 | 31.556 | 31.089 | 34.197 | 39.744 | 52.889 | 72.479 | 87.167 | 86.394 | 79.237 |
| 82 | Somalia | Sanaag | 31.924 | 28.802 | 28.341 | 31.180 | 36.890 | 47.579 | 62.852 | 71.950 | 70.594 | 68.187 |
| 83 | Somalia | Shabeellaha Dhexe | 19.796 | 21.649 | 25.923 | 32.318 | 41.570 | 57.057 | 77.907 | 97.955 | 108.501 | 116.098 |
| 84 | Somalia | Shabeellaha Hoose | 17.812 | 20.914 | 27.641 | 37.554 | 52.826 | 76.494 | 105.153 | 129.922 | 138.832 | 141.318 |
| 85 | Somalia | Sool | 34.141 | 30.473 | 30.056 | 33.424 | 39.781 | 52.788 | 72.060 | 85.212 | 84.169 | 79.610 |
| 86 | Somalia | Togdheer | 55.959 | 42.952 | 35.129 | 33.650 | 38.420 | 50.189 | 68.875 | 81.590 | 82.124 | 79.332 |
| 87 | Somalia | Woqooyi Galbeed | 45.031 | 33.666 | 25.760 | 24.452 | 28.458 | 39.558 | 58.153 | 71.401 | 76.724 | 76.000 |
| 88 | South Sudan | Central Equatoria | 485.838 | 464.997 | 425.742 | 381.944 | 360.892 | 354.233 | 372.940 | 374.183 | 409.856 | 401.786 |
| 89 | South Sudan | Eastern Equatoria | 361.644 | 365.467 | 370.234 | 352.710 | 342.706 | 337.645 | 346.149 | 334.763 | 355.233 | 331.106 |
| 90 | South Sudan | Jungoli | 191.880 | 202.534 | 218.515 | 222.060 | 220.571 | 211.188 | 215.461 | 212.211 | 235.797 | 233.158 |
| 91 | South Sudan | Lakes | 275.409 | 264.393 | 258.021 | 247.970 | 241.556 | 234.031 | 246.050 | 249.301 | 286.164 | 287.232 |
| 92 | South Sudan | North Bahr-al-Ghazal | 205.873 | 205.198 | 212.134 | 213.303 | 210.104 | 201.474 | 203.536 | 199.843 | 220.955 | 217.175 |
| 93 | South Sudan | Unity | 156.672 | 156.875 | 164.265 | 168.614 | 169.903 | 168.740 | 179.084 | 181.598 | 210.823 | 211.606 |
| 94 | South Sudan | Upper Nile | 139.745 | 158.538 | 184.186 | 195.374 | 195.383 | 184.396 | 183.605 | 178.197 | 195.555 | 194.102 |
| 95 | South Sudan | Warap | 199.671 | 194.304 | 195.966 | 194.846 | 192.440 | 188.257 | 198.809 | 201.500 | 232.711 | 232.471 |
| 96 | South Sudan | West Bahr-al-Ghazal | 328.350 | 321.021 | 313.315 | 301.440 | 290.763 | 281.590 | 291.098 | 292.061 | 327.437 | 327.157 |
| 97 | South Sudan | West Equatoria | 491.131 | 477.066 | 455.807 | 428.471 | 412.703 | 401.849 | 412.766 | 409.962 | 455.398 | 456.670 |
| 98 | Sudan | Al Jazirah | 21.450 | 18.974 | 16.617 | 16.748 | 19.426 | 25.849 | 35.809 | 44.999 | 54.815 | 62.833 |
| 99 | Sudan | Al Qadarif | 43.771 | 43.167 | 41.989 | 41.345 | 39.179 | 40.623 | 45.652 | 47.689 | 52.905 | 58.947 |
| 100 | Sudan | Blue Nile | 70.231 | 55.797 | 47.025 | 45.677 | 48.691 | 58.714 | 74.507 | 83.265 | 92.919 | 99.610 |
| 101 | Sudan | Central Darfur | 61.801 | 62.121 | 68.238 | 75.570 | 79.018 | 81.199 | 85.282 | 85.974 | 91.359 | 96.771 |
| 102 | Sudan | East Darfur | 39.237 | 36.641 | 36.890 | 41.322 | 48.413 | 57.439 | 70.221 | 78.985 | 88.783 | 97.145 |
| 103 | Sudan | Kassala | 37.768 | 44.504 | 50.015 | 53.684 | 52.728 | 51.456 | 52.375 | 51.929 | 55.359 | 61.775 |
| 104 | Sudan | Khartoum | 16.915 | 17.815 | 16.871 | 17.329 | 19.366 | 20.602 | 23.959 | 25.973 | 29.263 | 36.197 |
| 105 | Sudan | North Darfur | 28.737 | 32.408 | 40.282 | 48.975 | 56.447 | 59.157 | 62.247 | 68.598 | 70.305 | 76.213 |
| 106 | Sudan | North Kurdufan | 34.297 | 27.623 | 21.754 | 21.877 | 26.507 | 32.928 | 43.176 | 53.069 | 58.857 | 65.947 |
| 107 | Sudan | Northern | 8.698 | 6.711 | 6.366 | 7.326 | 10.108 | 11.958 | 13.421 | 16.101 | 16.534 | 19.198 |
| 108 | Sudan | Red Sea | 15.036 | 13.207 | 12.463 | 13.430 | 15.169 | 18.292 | 24.127 | 27.525 | 31.970 | 36.257 |
| 109 | Sudan | River Nile | 12.927 | 11.564 | 10.229 | 10.576 | 13.316 | 14.124 | 18.459 | 22.209 | 25.448 | 32.517 |
| 110 | Sudan | Sennar | 24.374 | 26.309 | 28.319 | 32.643 | 37.143 | 44.848 | 55.583 | 63.298 | 73.078 | 77.533 |
| 111 | Sudan | South Darfur | 52.435 | 62.156 | 76.761 | 87.600 | 90.730 | 86.581 | 84.756 | 83.872 | 89.273 | 98.035 |
| 112 | Sudan | South Kurdufan | 34.579 | 34.126 | 36.413 | 41.883 | 47.760 | 55.810 | 66.956 | 72.422 | 80.334 | 85.516 |
| 113 | Sudan | West Darfur | 44.148 | 41.828 | 44.198 | 49.509 | 55.800 | 62.160 | 71.311 | 76.794 | 81.277 | 83.003 |
| 114 | Sudan | West Kurdufan | 42.606 | 48.128 | 56.693 | 67.523 | 74.505 | 78.555 | 81.747 | 83.624 | 87.394 | 96.236 |
| 115 | Sudan | White Nile | 25.692 | 24.334 | 24.058 | 27.083 | 31.659 | 39.819 | 51.805 | 58.792 | 65.181 | 70.014 |

**RID**: Regional Identification number

**Table S3** Observed value of the *P.vivax* yearly malaria incidence rates

| **RID** | **Country** | **Region** | ***P. vivax* yearly malaria incidence rates** | | | | | | | | | |
| --- | --- | --- | --- | --- | --- | --- | --- | --- | --- | --- | --- | --- |
|  |  |  | **2011** | **2012** | **2013** | **2014** | **2015** | **2016** | **2017** | **2018** | **2019** | **2020** |
| 1 | Djibouti | Ali Sabieh | 0.002 | 0.000 | 0.076 | 0.638 | 0.567 | 1.407 | 3.760 | 5.560 | 8.273 | 17.410 |
| 2 | Djibouti | Dikhil | 0.001 | 0.000 | 0.055 | 0.460 | 0.390 | 0.782 | 1.838 | 2.831 | 4.504 | 11.549 |
| 3 | Djibouti | Djibouti | 0.000 | 0.000 | 0.002 | 0.018 | 0.017 | 0.053 | 0.148 | 0.243 | 0.348 | 0.743 |
| 4 | Djibouti | Obock | 0.066 | 0.000 | 1.703 | 13.071 | 11.612 | 30.627 | 82.389 | 131.235 | 183.922 | 355.614 |
| 5 | Djibouti | Tadjourah | 0.045 | 0.012 | 1.243 | 10.579 | 9.896 | 26.070 | 68.203 | 107.700 | 152.120 | 293.514 |
| 6 | Eritrea | Anseba | 4.056 | 3.200 | 1.515 | 1.820 | 1.416 | 0.987 | 3.275 | 2.766 | 3.441 | 1.515 |
| 7 | Eritrea | Debub | 6.165 | 4.357 | 1.776 | 1.960 | 1.495 | 1.087 | 3.751 | 3.305 | 4.238 | 1.872 |
| 8 | Eritrea | Debubawi Keyih Bahri | 6.580 | 6.006 | 3.349 | 4.385 | 3.324 | 2.291 | 7.675 | 6.516 | 7.279 | 2.873 |
| 9 | Eritrea | Gash Barka | 8.757 | 10.261 | 6.409 | 8.581 | 6.069 | 3.180 | 7.492 | 5.140 | 5.741 | 2.485 |
| 10 | Eritrea | Maekel | 3.216 | 2.262 | 0.970 | 1.135 | 0.864 | 0.596 | 1.906 | 1.628 | 2.027 | 0.922 |
| 11 | Eritrea | Semenawi Keyih Bahri | 4.642 | 3.314 | 1.469 | 1.745 | 1.384 | 1.023 | 3.591 | 3.157 | 3.979 | 1.754 |
| 12 | Ethiopia | Addis Abeba | 5.503 | 8.083 | 6.949 | 4.038 | 3.033 | 1.680 | 0.857 | 0.161 | 0.194 | 0.279 |
| 13 | Ethiopia | Afar | 64.479 | 51.404 | 31.113 | 15.897 | 12.220 | 9.326 | 8.527 | 2.692 | 4.222 | 3.484 |
| 14 | Ethiopia | Amhara | 99.828 | 56.606 | 23.412 | 9.627 | 6.717 | 5.384 | 5.404 | 1.784 | 2.964 | 4.626 |
| 15 | Ethiopia | Benshangul-Gumaz | 97.577 | 98.932 | 73.779 | 43.401 | 37.235 | 31.780 | 28.713 | 7.487 | 10.220 | 4.934 |
| 16 | Ethiopia | Dire Dawa | 67.228 | 94.166 | 79.587 | 45.048 | 32.417 | 18.185 | 9.623 | 1.966 | 2.614 | 6.235 |
| 17 | Ethiopia | Gambela Peoples | 92.566 | 97.695 | 70.555 | 41.776 | 37.436 | 33.342 | 32.760 | 8.959 | 11.928 | 6.352 |
| 18 | Ethiopia | Harari People | 65.083 | 91.814 | 77.097 | 42.423 | 29.258 | 14.529 | 6.785 | 1.334 | 1.839 | 6.656 |
| 19 | Ethiopia | Oromia | 50.231 | 87.669 | 85.489 | 50.746 | 40.530 | 26.021 | 15.339 | 3.268 | 4.191 | 8.218 |
| 20 | Ethiopia | Somali | 50.172 | 47.800 | 35.749 | 21.627 | 19.105 | 16.792 | 16.786 | 5.287 | 7.814 | 11.030 |
| 21 | Ethiopia | Southern Nations, Nationalities and Peoples | 69.594 | 70.923 | 51.197 | 29.080 | 23.660 | 17.800 | 14.931 | 4.074 | 5.791 | 14.793 |
| 22 | Ethiopia | Tigray | 82.606 | 46.306 | 19.256 | 8.344 | 6.507 | 6.713 | 8.478 | 2.927 | 4.557 | 16.250 |
| 23 | Kenya | Baringo | 0.000 | 0.000 | 0.000 | 0.000 | 0.000 | 0.000 | 0.000 | 0.000 | 0.000 | 0.000 |
| 24 | Kenya | Bomet | 0.000 | 0.000 | 0.000 | 0.000 | 0.000 | 0.000 | 0.000 | 0.000 | 0.000 | 0.000 |
| 25 | Kenya | Bungoma | 0.000 | 0.000 | 0.000 | 0.000 | 0.000 | 0.000 | 0.000 | 0.000 | 0.000 | 0.000 |
| 26 | Kenya | Busia | 0.000 | 0.000 | 0.000 | 0.000 | 0.000 | 0.000 | 0.000 | 0.000 | 0.000 | 0.000 |
| 27 | Kenya | Elgeyo-Marakwet | 0.000 | 0.000 | 0.000 | 0.000 | 0.000 | 0.000 | 0.000 | 0.000 | 0.000 | 0.000 |
| 28 | Kenya | Embu | 0.000 | 0.000 | 0.000 | 0.000 | 0.000 | 0.000 | 0.000 | 0.000 | 0.000 | 0.000 |
| 29 | Kenya | Garissa | 0.000 | 0.000 | 0.000 | 0.000 | 0.000 | 0.000 | 0.000 | 0.000 | 0.000 | 0.000 |
| 30 | Kenya | Homa Bay | 0.000 | 0.000 | 0.000 | 0.000 | 0.000 | 0.000 | 0.000 | 0.000 | 0.000 | 0.000 |
| 31 | Kenya | Isiolo | 0.000 | 0.000 | 0.000 | 0.000 | 0.000 | 0.000 | 0.000 | 0.000 | 0.000 | 0.000 |
| 32 | Kenya | Kajiado | 0.000 | 0.000 | 0.000 | 0.000 | 0.000 | 0.000 | 0.000 | 0.000 | 0.000 | 0.000 |
| 33 | Kenya | Kakamega | 0.000 | 0.000 | 0.000 | 0.000 | 0.000 | 0.000 | 0.000 | 0.000 | 0.000 | 0.000 |
| 34 | Kenya | Kericho | 0.000 | 0.000 | 0.000 | 0.000 | 0.000 | 0.000 | 0.000 | 0.000 | 0.000 | 0.000 |
| 35 | Kenya | Kiambu | 0.000 | 0.000 | 0.000 | 0.000 | 0.000 | 0.000 | 0.000 | 0.000 | 0.000 | 0.000 |
| 36 | Kenya | Kilifi | 0.000 | 0.000 | 0.000 | 0.000 | 0.000 | 0.000 | 0.000 | 0.000 | 0.000 | 0.000 |
| 37 | Kenya | Kirinyaga | 0.000 | 0.000 | 0.000 | 0.000 | 0.000 | 0.000 | 0.000 | 0.000 | 0.000 | 0.000 |
| 38 | Kenya | Kisii | 0.000 | 0.000 | 0.000 | 0.000 | 0.000 | 0.000 | 0.000 | 0.000 | 0.000 | 0.000 |
| 39 | Kenya | Kisumu | 0.000 | 0.000 | 0.000 | 0.000 | 0.000 | 0.000 | 0.000 | 0.000 | 0.000 | 0.000 |
| 40 | Kenya | Kitui | 0.000 | 0.000 | 0.000 | 0.000 | 0.000 | 0.000 | 0.000 | 0.000 | 0.000 | 0.000 |
| 41 | Kenya | Kwale | 0.000 | 0.000 | 0.000 | 0.000 | 0.000 | 0.000 | 0.000 | 0.000 | 0.000 | 0.000 |
| 42 | Kenya | Laikipia | 0.000 | 0.000 | 0.000 | 0.000 | 0.000 | 0.000 | 0.000 | 0.000 | 0.000 | 0.000 |
| 43 | Kenya | Lamu | 0.000 | 0.000 | 0.000 | 0.000 | 0.000 | 0.000 | 0.000 | 0.000 | 0.000 | 0.000 |
| 44 | Kenya | Machakos | 0.000 | 0.000 | 0.000 | 0.000 | 0.000 | 0.000 | 0.000 | 0.000 | 0.000 | 0.000 |
| 45 | Kenya | Makueni | 0.000 | 0.000 | 0.000 | 0.000 | 0.000 | 0.000 | 0.000 | 0.000 | 0.000 | 0.000 |
| 46 | Kenya | Mandera | 0.000 | 0.000 | 0.000 | 0.000 | 0.000 | 0.000 | 0.000 | 0.000 | 0.000 | 0.000 |
| 47 | Kenya | Marsabit | 0.000 | 0.000 | 0.000 | 0.000 | 0.000 | 0.000 | 0.000 | 0.000 | 0.000 | 0.000 |
| 48 | Kenya | Meru | 0.000 | 0.000 | 0.000 | 0.000 | 0.000 | 0.000 | 0.000 | 0.000 | 0.000 | 0.000 |
| 49 | Kenya | Migori | 0.000 | 0.000 | 0.000 | 0.000 | 0.000 | 0.000 | 0.000 | 0.000 | 0.000 | 0.000 |
| 50 | Kenya | Mombasa | 0.000 | 0.000 | 0.000 | 0.000 | 0.000 | 0.000 | 0.000 | 0.000 | 0.000 | 0.000 |
| 51 | Kenya | Murang'a | 0.000 | 0.000 | 0.000 | 0.000 | 0.000 | 0.000 | 0.000 | 0.000 | 0.000 | 0.000 |
| 52 | Kenya | Nairobi | 0.000 | 0.000 | 0.000 | 0.000 | 0.000 | 0.000 | 0.000 | 0.000 | 0.000 | 0.000 |
| 53 | Kenya | Nakuru | 0.000 | 0.000 | 0.000 | 0.000 | 0.000 | 0.000 | 0.000 | 0.000 | 0.000 | 0.000 |
| 54 | Kenya | Nandi | 0.000 | 0.000 | 0.000 | 0.000 | 0.000 | 0.000 | 0.000 | 0.000 | 0.000 | 0.000 |
| 55 | Kenya | Narok | 0.000 | 0.000 | 0.000 | 0.000 | 0.000 | 0.000 | 0.000 | 0.000 | 0.000 | 0.000 |
| 56 | Kenya | Nyamira | 0.000 | 0.000 | 0.000 | 0.000 | 0.000 | 0.000 | 0.000 | 0.000 | 0.000 | 0.000 |
| 57 | Kenya | Nyandarua | 0.000 | 0.000 | 0.000 | 0.000 | 0.000 | 0.000 | 0.000 | 0.000 | 0.000 | 0.000 |
| 58 | Kenya | Nyeri | 0.000 | 0.000 | 0.000 | 0.000 | 0.000 | 0.000 | 0.000 | 0.000 | 0.000 | 0.000 |
| 59 | Kenya | Samburu | 0.000 | 0.000 | 0.000 | 0.000 | 0.000 | 0.000 | 0.000 | 0.000 | 0.000 | 0.000 |
| 60 | Kenya | Siaya | 0.000 | 0.000 | 0.000 | 0.000 | 0.000 | 0.000 | 0.000 | 0.000 | 0.000 | 0.000 |
| 61 | Kenya | Taita Taveta | 0.000 | 0.000 | 0.000 | 0.000 | 0.000 | 0.000 | 0.000 | 0.000 | 0.000 | 0.000 |
| 62 | Kenya | Tana River | 0.000 | 0.000 | 0.000 | 0.000 | 0.000 | 0.000 | 0.000 | 0.000 | 0.000 | 0.000 |
| 63 | Kenya | Tharaka-Nithi | 0.000 | 0.000 | 0.000 | 0.000 | 0.000 | 0.000 | 0.000 | 0.000 | 0.000 | 0.000 |
| 64 | Kenya | Trans Nzoia | 0.000 | 0.000 | 0.000 | 0.000 | 0.000 | 0.000 | 0.000 | 0.000 | 0.000 | 0.000 |
| 65 | Kenya | Turkana | 0.000 | 0.000 | 0.000 | 0.000 | 0.000 | 0.000 | 0.000 | 0.000 | 0.000 | 0.000 |
| 66 | Kenya | Uasin Gishu | 0.000 | 0.000 | 0.000 | 0.000 | 0.000 | 0.000 | 0.000 | 0.000 | 0.000 | 0.000 |
| 67 | Kenya | Vihiga | 0.000 | 0.000 | 0.000 | 0.000 | 0.000 | 0.000 | 0.000 | 0.000 | 0.000 | 0.000 |
| 68 | Kenya | Wajir | 0.000 | 0.000 | 0.000 | 0.000 | 0.000 | 0.000 | 0.000 | 0.000 | 0.000 | 0.000 |
| 69 | Kenya | West Pokot | 0.000 | 0.000 | 0.000 | 0.000 | 0.000 | 0.000 | 0.000 | 0.000 | 0.000 | 0.000 |
| 70 | Somalia | Awdal | 0.000 | 0.360 | 0.363 | 0.384 | 0.422 | 0.490 | 0.604 | 0.693 | 5.956 | 0.824 |
| 71 | Somalia | Bakool | 0.000 | 0.297 | 0.414 | 0.569 | 0.789 | 1.094 | 1.445 | 1.755 | 14.402 | 1.819 |
| 72 | Somalia | Banaadir | 0.000 | 0.264 | 0.263 | 0.304 | 0.386 | 0.574 | 0.841 | 1.074 | 9.622 | 1.258 |
| 73 | Somalia | Bari | 0.000 | 0.411 | 0.408 | 0.467 | 0.562 | 0.757 | 1.035 | 1.221 | 9.621 | 1.168 |
| 74 | Somalia | Bay | 0.000 | 0.253 | 0.361 | 0.517 | 0.749 | 1.090 | 1.466 | 1.797 | 15.061 | 1.904 |
| 75 | Somalia | Galguduud | 0.000 | 0.317 | 0.315 | 0.337 | 0.381 | 0.485 | 0.644 | 0.787 | 6.400 | 0.817 |
| 76 | Somalia | Gedo | 0.000 | 0.232 | 0.332 | 0.479 | 0.691 | 1.010 | 1.362 | 1.668 | 13.648 | 1.699 |
| 77 | Somalia | Hiiraan | 0.000 | 0.416 | 0.447 | 0.505 | 0.613 | 0.809 | 1.043 | 1.256 | 10.087 | 1.278 |
| 78 | Somalia | Jubbada Dhexe | 0.000 | 0.353 | 0.484 | 0.663 | 0.887 | 1.223 | 1.597 | 1.863 | 15.098 | 1.817 |
| 79 | Somalia | Jubbada Hoose | 0.000 | 0.434 | 0.480 | 0.588 | 0.754 | 1.066 | 1.455 | 1.710 | 13.439 | 1.551 |
| 80 | Somalia | Mudug | 0.000 | 0.340 | 0.362 | 0.415 | 0.491 | 0.633 | 0.819 | 0.961 | 7.645 | 0.947 |
| 81 | Somalia | Nugaal | 0.000 | 0.374 | 0.369 | 0.406 | 0.472 | 0.627 | 0.855 | 1.024 | 8.072 | 0.955 |
| 82 | Somalia | Sanaag | 0.000 | 0.337 | 0.333 | 0.367 | 0.436 | 0.565 | 0.750 | 0.856 | 6.625 | 0.816 |
| 83 | Somalia | Shabeellaha Dhexe | 0.000 | 0.254 | 0.303 | 0.379 | 0.490 | 0.678 | 0.927 | 1.163 | 10.136 | 1.357 |
| 84 | Somalia | Shabeellaha Hoose | 0.000 | 0.247 | 0.327 | 0.444 | 0.624 | 0.900 | 1.233 | 1.521 | 12.810 | 1.625 |
| 85 | Somalia | Sool | 0.000 | 0.361 | 0.358 | 0.399 | 0.475 | 0.629 | 0.854 | 1.007 | 7.896 | 0.957 |
| 86 | Somalia | Togdheer | 0.000 | 0.506 | 0.417 | 0.400 | 0.457 | 0.597 | 0.819 | 0.974 | 7.821 | 0.961 |
| 87 | Somalia | Woqooyi Galbeed | 0.000 | 0.396 | 0.303 | 0.289 | 0.337 | 0.470 | 0.695 | 0.855 | 7.268 | 0.911 |
| 88 | South Sudan | Central Equatoria | 0.000 | 0.000 | 0.000 | 0.000 | 0.000 | 0.000 | 0.000 | 0.000 | 0.000 | 0.000 |
| 89 | South Sudan | Eastern Equatoria | 0.000 | 0.000 | 0.000 | 0.000 | 0.000 | 0.000 | 0.000 | 0.000 | 0.000 | 0.000 |
| 90 | South Sudan | Jungoli | 0.000 | 0.000 | 0.000 | 0.000 | 0.000 | 0.000 | 0.000 | 0.000 | 0.000 | 0.000 |
| 91 | South Sudan | Lakes | 0.000 | 0.000 | 0.000 | 0.000 | 0.000 | 0.000 | 0.000 | 0.000 | 0.000 | 0.000 |
| 92 | South Sudan | North Bahr-al-Ghazal | 0.000 | 0.000 | 0.000 | 0.000 | 0.000 | 0.000 | 0.000 | 0.000 | 0.000 | 0.000 |
| 93 | South Sudan | Unity | 0.000 | 0.000 | 0.000 | 0.000 | 0.000 | 0.000 | 0.000 | 0.000 | 0.000 | 0.000 |
| 94 | South Sudan | Upper Nile | 0.000 | 0.000 | 0.000 | 0.000 | 0.000 | 0.000 | 0.000 | 0.000 | 0.000 | 0.000 |
| 95 | South Sudan | Warap | 0.000 | 0.000 | 0.000 | 0.000 | 0.000 | 0.000 | 0.000 | 0.000 | 0.000 | 0.000 |
| 96 | South Sudan | West Bahr-al-Ghazal | 0.000 | 0.000 | 0.000 | 0.000 | 0.000 | 0.000 | 0.000 | 0.000 | 0.000 | 0.000 |
| 97 | South Sudan | West Equatoria | 0.000 | 0.000 | 0.000 | 0.000 | 0.000 | 0.000 | 0.000 | 0.000 | 0.000 | 0.000 |
| 98 | Sudan | Al Jazirah | 3.629 | 3.209 | 2.828 | 2.869 | 3.321 | 3.420 | 3.446 | 4.700 | 7.324 | 7.900 |
| 99 | Sudan | Al Qadarif | 7.365 | 7.271 | 7.116 | 7.069 | 6.773 | 5.608 | 4.719 | 5.381 | 7.544 | 7.792 |
| 100 | Sudan | Blue Nile | 11.911 | 9.424 | 7.840 | 7.548 | 8.039 | 7.708 | 7.367 | 9.115 | 13.145 | 13.421 |
| 101 | Sudan | Central Darfur | 10.450 | 10.478 | 11.436 | 12.643 | 13.284 | 10.941 | 8.783 | 9.855 | 13.354 | 12.984 |
| 102 | Sudan | East Darfur | 6.580 | 6.177 | 6.219 | 6.940 | 8.105 | 7.605 | 7.011 | 8.815 | 12.888 | 13.248 |
| 103 | Sudan | Kassala | 6.348 | 7.494 | 8.427 | 9.035 | 8.833 | 6.779 | 5.182 | 5.694 | 7.810 | 8.205 |
| 104 | Sudan | Khartoum | 2.857 | 3.003 | 2.850 | 2.936 | 3.267 | 2.698 | 2.284 | 2.693 | 3.877 | 4.520 |
| 105 | Sudan | North Darfur | 4.845 | 5.456 | 6.739 | 8.162 | 9.448 | 8.031 | 6.583 | 8.088 | 10.507 | 10.345 |
| 106 | Sudan | North Kurdufan | 5.809 | 4.683 | 3.706 | 3.742 | 4.550 | 4.497 | 4.453 | 6.065 | 8.619 | 9.046 |
| 107 | Sudan | Northern | 1.473 | 1.127 | 1.063 | 1.223 | 1.697 | 1.631 | 1.427 | 1.923 | 2.514 | 2.705 |
| 108 | Sudan | Red Sea | 2.535 | 2.233 | 2.115 | 2.281 | 2.593 | 2.527 | 2.571 | 3.267 | 4.820 | 5.100 |
| 109 | Sudan | River Nile | 2.187 | 1.947 | 1.720 | 1.775 | 2.225 | 1.865 | 1.832 | 2.432 | 3.563 | 4.309 |
| 110 | Sudan | Sennar | 4.124 | 4.457 | 4.821 | 5.577 | 6.350 | 6.021 | 5.499 | 6.838 | 10.188 | 10.347 |
| 111 | Sudan | South Darfur | 8.824 | 10.493 | 12.959 | 14.748 | 15.214 | 11.394 | 8.341 | 9.212 | 12.815 | 13.326 |
| 112 | Sudan | South Kurdufan | 5.859 | 5.786 | 6.206 | 7.166 | 8.185 | 7.585 | 6.880 | 8.271 | 11.776 | 11.743 |
| 113 | Sudan | West Darfur | 7.501 | 7.088 | 7.441 | 8.331 | 9.450 | 8.405 | 7.242 | 8.551 | 11.530 | 11.010 |
| 114 | Sudan | West Kurdufan | 7.193 | 8.133 | 9.573 | 11.434 | 12.697 | 10.724 | 8.526 | 9.786 | 13.203 | 13.541 |
| 115 | Sudan | White Nile | 4.337 | 4.104 | 4.083 | 4.617 | 5.394 | 5.334 | 5.162 | 6.421 | 9.082 | 9.136 |

**RID**: Regional Identification number
